# Using Variational Multi-view Learning for Classification of Grocery Items

**DOI:** 10.1016/j.patter.2020.100143

**Published:** 2020-11-13

**Authors:** Marcus Klasson, Cheng Zhang, Hedvig Kjellström

**Affiliations:** 1 Division of Robotics, Perception, and Learning, Lindstedtsvägen 24, 114 28 Stockholm, Sweden; 2 Microsoft Research Ltd, 21 Station Road, Cambridge CB1 2FB, UK

**Keywords:** DSML 2: Proof-of-Concept: Data science output has been formulated, implemented, and tested for one domain/problem

## Abstract

An essential task for computer vision-based assistive technologies is to help visually impaired people to recognize objects in constrained environments, for instance, recognizing food items in grocery stores. In this paper, we introduce a novel dataset with natural images of groceries—fruits, vegetables, and packaged products—where all images have been taken inside grocery stores to resemble a shopping scenario. Additionally, we download iconic images and text descriptions for each item that can be utilized for better representation learning of groceries. We select a multi-view generative model, which can combine the different item information into lower-dimensional representations. The experiments show that utilizing the additional information yields higher accuracies on classifying grocery items than only using the natural images. We observe that iconic images help to construct representations separated by visual differences of the items, while text descriptions enable the model to distinguish between visually similar items by different ingredients.

## Highlights

• Introducing a dataset of grocery items with real images and web-scraped information
• Best model performance is achieved when all information in the dataset is combined
• Web-scraped images help to group the grocery items based on their color and shape
• Web-scraped text separates groceries based on differences in ingredients and flavor

## The Bigger Picture

In recent years, several computer vision-based assistive technologies for helping visually impaired people have been released on the market. We study a special case whereby visual capability is often important when searching for objects: grocery shopping. To enable assistive vision devices for grocery shopping, data representing the grocery items have to be available. We, therefore, provide a challenging dataset of smartphone images of grocery items resembling the shopping scenario with an assistive vision device. Our dataset is publicly available to encourage other researchers to evaluate their computer vision models on grocery item classification in real-world environments. The next step would be to deploy the trained models into mobile devices, such as smartphone applications, to evaluate whether the models can perform effectively in real time via human users. This dataset is a step toward enabling these technologies to make everyday life easier for the visually impaired.

## Teaser

Assistive vision devices are used for recognizing food items to help visually impaired people with grocery shopping on their own. We have collected smartphone images of groceries using an assistive device when shopping. Our results show that utilizing both smartphone images and data downloaded from supermarket websites can enhance a system's grocery recognition capabilities compared with only using smartphone images. Our study is thus a step toward fully image-based assistive devices for helping the visually impaired with grocery shopping.

## Introduction

In recent years, computer vision-based assistive technologies have been developed for supporting people with visual impairments. Such technologies exist in the form of mobile applications, e.g., Microsoft's Seeing AI (https://www.microsoft.com/en-us/seeing-ai/) and Aipoly Vision (https://www.aipoly.com/), and as wearable artificial vision devices, e.g., Orcam MyEye (https://www.orcam.com/en/), Transsense (https://www.transsense.ai/), and the Sound of Vision system.^1^ These products can support people with visual impairments in many different situations, such as reading text documents, describing the user's environment, and recognizing people the user may know. In this paper, we focus on an application that is essential for assistive vision, namely visual support when shopping for grocery items, considering a large range of edible items including fruits, vegetables, and refrigerated products, e.g., milk and juice packages.

**Figure 1:**
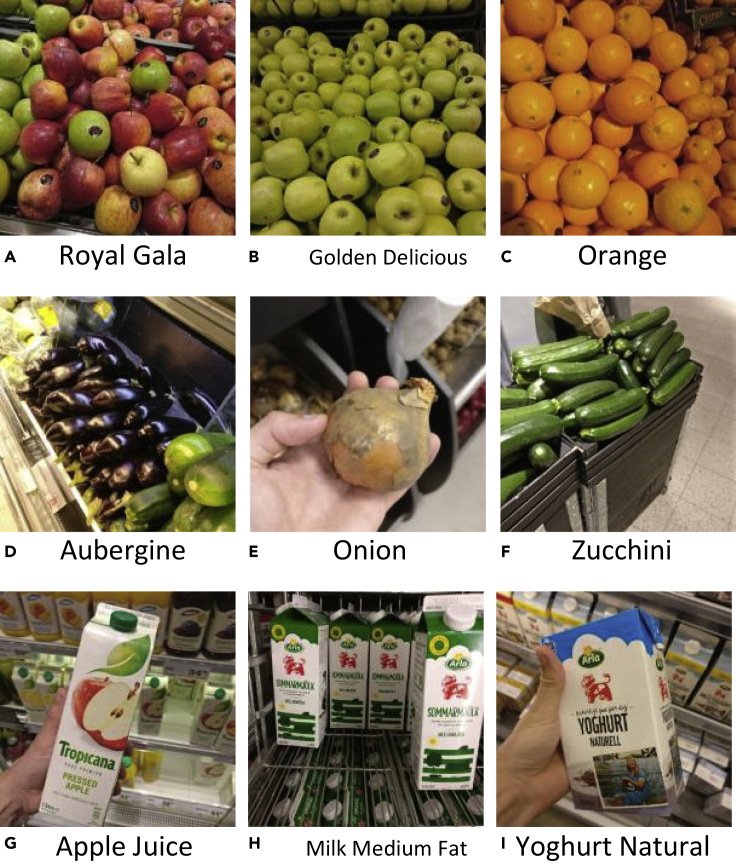
Examples of Natural Images in Our Dataset, for which Each Image Has Been Taken inside a Grocery Store Image examples of fruits, vegetables, and refrigerated products are presented in top, middle, and bottom rows, respectively.

Grocery shopping with low vision capabilities can be difficult for various reasons. For example, in grocery store sections for raw groceries, the items are often stacked in large bins as shown in Figures 1A–1F. Additionally, similar items are usually stacked next to each other, so that can be misplaced into neighboring bins. Humans can distinguish between groceries without vision to some degree, e.g., by touch and smell, but this requires prior knowledge about texture and fragrance of food items. Furthermore, packaged items, e.g., milk, juice, and yogurt cartons, can only be differentiated with the help of visual information (see Figures 1G–1I). Such items usually have barcodes that are readable using the existing assistive vision devices described above. Although using a barcode detector is a clever solution, it can be inconvenient and exhausting always having to detect barcodes of packaged items. Therefore, an assistive vision device that relies on natural images would be of significant value for a visually impaired person in a grocery store.

For an image classification model to be capable of recognizing grocery items in the setting of grocery shopping, we need image data from grocery store environments. In our previous work,^2^ we addressed this by collecting a novel dataset containing natural images of various raw grocery items and refrigerated products, where all images were taken with a smartphone camera inside grocery stores to simulate a realistic shopping scenario. In addition to the natural images, we also looked at alternative types of grocery item data that could facilitate the classification task. Grocery store chains commonly maintain websites where they store information about the grocery items they sell and are currently available in the store for online grocery shopping. For every item, there is usually an iconic image of the item on a white background, a text description about the item, and information about nutrition values, origin country, and so forth. We downloaded such information from an online grocery shopping website to complement the natural images in the dataset.

To utilize the available information from the dataset for grocery item classification, we use a multi-view generative model, Variational Canonical Correlation Analysis (VCCA),^3^ which learns a shared representation of the natural images and the downloaded information. A view can be defined as any signal or data measured by some appropriate sensor, and combining information from multiple views has previously been shown to be helpful for various image classification tasks.^4^, ^5^, ^6^, ^7^, ^8^, ^9^, ^10^, ^11^ However, naively adding more additional information may not lead to improved results and even harm the performance of the model.^12^^,^^13^ We, therefore, perform an ablation study over the available views in the dataset with VCCA to gain insights into how each view can affect the classification performance. Moreover, VCCA allows separation of the latent space into shared and private components, where the private latent spaces should hold information about a single view. This might prove useful for reducing view-specific noise in the shared latent space, which can ease the learning process of the representations we want to use for classification. We investigate the effects each view has on the learned representations of VCCA by measuring classification performances as well as visualizing the latent space for the different model settings. The contributions of this paper are as the follows.

We present a novel dataset with natural images of grocery items as well as iconic images and text descriptions for every item class (see The Grocery Store Dataset in Results).^2^ The natural images are taken in grocery stores in different lighting conditions and backgrounds that can be challenging settings for a computer vision-based assistive device. The additional iconic images and text descriptions make the dataset suitable for multi-view learning by combining the natural images with the extra information to obtain better classification performance.

We investigate how to use information from different views for the task of grocery item classification with the deep generative model, VCCA (see Methods in Experimental Procedures). This model combines information from multiple views into a low-dimensional latent representation that can be used for classification. We also select a variant of VCCA, denoted VCCA-private, which separates shared and private information about each view through factorization of the latent representation (see Extracting Private Information of Views with VCCA-private in Experimental Procedures). Furthermore, we use a standard multi-view autoencoder model called Split Autoencoder (SplitAE)^13^^,^^14^ for benchmarking against VCCA and VCCA-private on classification.

We conduct experiments with SplitAE, VCCA, and VCCA-private on the task of grocery item classification with our dataset (Results). We perform a thorough ablation study over all views in the dataset to demonstrate how each view contributes to enhancing the classification performance and conclude that utilizing the web-scraped views yields better classification results than only using the natural images (see Classification Results in Results). To gain further insights into the results, we visualize the learned latent representations of the VCCA models and discuss how the iconic images and textual descriptions impose different structures on the latent space that are beneficial for classification (see Investigation of the Learned Representations in Results).

This work is an extended version of Klasson et al.,^2^ in which we first presented this dataset. In this paper, we have added a validation set of natural images from two stores that were not present in the training and test set splits from Klasson et al.^2^ to avoid overfitting effects. We also demonstrate how the text descriptions can be utilized alone and along with the iconic images in a multi-view setting, while Klasson et al.^2^ only experimented with the combination of natural and iconic images to build better representations of grocery items. Finally, we decode iconic images from unseen natural images as an alternative to evaluate the usefulness of the latent representations (see Decoding Iconic Images from Unseen Natural Images in Results). As we only evaluated the decoded iconic images qualitatively in Klasson et al.,^2^ we have extended the assessment by comparing the quality of the decoded images from different VCCA models with multiple image similarity metrics.

Next we discuss image datasets, food datasets, and multi-view models related to our work:

### Image Datasets

Many image datasets used in computer vision have been collected by downloading images from the web.^15^, ^16^, ^17^, ^18^, ^19^, ^20^, ^21^, ^22^, ^23^, ^24^, ^25^, ^26^ Some datasets^15^^,^^18^^,^^20^^,^^22^^,^^24^ use search words with the object category in isolation, which typically returns high-quality images where the searched object is large and centered. To collect images from more real-world scenarios, searching for combinations of object categories usually returns images of two searched categories but also numerous other categories.^21^^,^^26^ The simplest annotation of these images is to provide a class label for the present objects. Occasionally, the dataset can use a hierarchical labeling structure and provide a fine- and coarse-grained label to objects where it is applicable. The annotators can also be asked to increase the possible level of supervision for the objects by, for instance, providing bounding boxes, segmentation masks, keypoints, text captions that describe the scene, and reference images of the objects.^17^^,^^19^^,^^21^^,^^22^^,^^24^^,^^26^ Our dataset includes reference (iconic) images of the objects that were web-scraped from a grocery store website. We also downloaded text descriptions that describe general attributes of the grocery items, such as flavor and texture, rather than the whole visual scene. The grocery items have also been labeled hierarchically in a fine- and coarse-grained manner if there exist multiple variants of specific items. For example, fine-grained classes of apples such as Golden Delicious or Royal Gala belong to the coarse-grained class “Apple.”

### Food Datasets

Recognizing grocery items in their natural environments, such as grocery stores, shelves, and kitchens, have been addressed in numerous previous works.^2^^,^^27^, ^28^, ^29^, ^30^, ^31^, ^32^, ^33^, ^34^, ^35^, ^36^, ^37^ The addressed tasks range over hierarchical classification, object detection, segmentation, and three-dimensional (3D) model generation. Most of these works collect a dataset that resembles shopping or cooking scenarios, whereby the datasets vary in the degree of labeling, different camera views, and the data domain difference between the training and test set. The training sets in GroZi-120,^32^ Grocery Products,^28^ and CAPG-GP^27^ datasets were obtained by web-scraping product images of single instances on grocery web stores, while the test sets were collected in grocery stores where there can be single and multiple instances of the same item and other different items. The RPC^35^ and TGFS^37^ datasets are used for object detection and classification of grocery products, whereby RPC is targeted for automatic checkout systems and TGFS is given the task of recognizing items purchased from self-service vending machines. The BigBIRD^33^ dataset and datasets from Hsiao et al.^29^, ^31^ and Lai et al.^29^, ^31^ contain images of grocery items from multiple camera views, segmentation masks, and depth maps for 3D reconstruction of various items. The Freiburg Groceries^30^ dataset contains images taken with smartphone cameras of items inside grocery stores, while its test set consists of smartphone photos in home environments with single or multiple instances from different kinds of items. The dataset presented in Waltner et al.^34^ also contains images taken with smartphone cameras inside grocery stores to develop a mobile application for recognizing raw food items and provide details about the item, such as nutrition values and recommendations of similar items. Other works that collected datasets of raw food items, such as fruits and vegetables, focused on the standard image classification task^38^^,^^39^ and on detecting fruits in orchards for robotic harvesting.^40^^,^^41^ Our dataset—the Grocery Store dataset—shares many similarities with the aforementioned works, for instance, all images of groceries being taken in their natural environment, the hierarchical labeling of the classes, and the iconic product images for each item in the dataset. Additionally, we have provided a text description for each item that was web-scraped along with the iconic image. As most grocery item datasets only include packaged products, we have also collected images of different fruit and vegetable classes along with packages in our dataset.

Other examples of food datasets are those with images of food dishes, for which Min et al.^42^ provide a summary of existing benchmark food datasets. The contents of these datasets range from images of food dishes,^43^, ^44^, ^45^, ^46^ cooking videos,^47^ recipes,^48^, ^49^, ^50^ and restaurant-oriented information.^51^^,^^52^ One major difference between recognizing groceries and food dishes is the appearance of the object categories in the images. For instance, a fruit or vegetable is usually intact and present in the image, while ingredients that are significant for recognizing a food dish may not be visible at all depending on the recipe. However, raw food items and dishes share similarities in recognition, since they can appear with many different visual features in the images compared with packaged groceries, e.g., carton boxes, cans, and bottles, where the object classes have identical shape and texture. Another key difference is the natural environments and scenes where the images of grocery items and food dishes have been taken. Food dish images usually show the food on a plate placed on a table and, occasionally, with cutlery and a glass next to the plate. Images taken in grocery stores can cover many instances of the same item stacked close to each other in shelves, baskets, and refrigerators, while there can be multiple different kinds of items next to each other in a kitchen environment. To summarize, recognizing grocery items and food dishes are both challenging tasks because examples from the same category can look very different and also appear in various realistic settings in images.

### Multi-view Learning Models

There exist many multi-view learning approaches for data fusion of multiple features.^3^^,^^5^^,^^6^^,^^13^^,^^53^, ^54^, ^55^, ^56^, ^57^, ^58^, ^59^, ^60^, ^61^, ^62^, ^63^, ^64^, ^65^, ^66^ A common approach is to obtain a shared latent space for all views with the assumption that each view has been generated from this shared space.^64^ A popular example of this is approach is Canonical Correlation Analysis (CCA),^67^ which aims to project two sets of variables (views) into a lower-dimensional space so that the correlation between the projections is maximized. Similar methods propose maximizing other alignment objectives for embedding the views, such as ranking losses.^5^^,^^6^^,^^55^^,^^63^ There exist nonlinear extensions of CCA, e.g., Kernel CCA^68^ and Deep CCA,^69^ which optimize their nonlinear feature mappings based on the CCA objective. Deep Canonically Correlated Autoencoders (DCCAE)^14^ is a Deep CCA model with an autoencoding part, which aims to maximize the canonical correlation between the extracted features as well as reconstructing the input data. Removing the CCA objective reduces DCCAE to a standard multi-view autoencoder, e.g., Bimodal Autoencoders and Split Autoencoders (SplitAEs),^13^^,^^14^ which only aim to learn a representation that best reconstructs the input data. SplitAEs aim to reconstruct two views from a representation encoded from one of the views. This approach was empirically shown to work better than Bimodal Autoencoders by Ngiam et al.^13^ in situations where only a single view is present at both training and test times.

Variational CCA (VCCA)^3^ can be seen as an extension of CCA to deep generative models, but can also be described as a probabilistic version of SplitAEs. VCCA uses the amortized inference procedure from variational autoencoders (VAEs)^70^^,^^71^ to learn the shared latent space by maximizing a lower bound on the data log likelihood of the views. Succeeding works have proposed new learning objectives and inference methods for enabling conditional generation of views, e.g., generating an image conditioned on some text and vice versa.^54^^,^^58^^,^^59^^,^^61^^,^^62^ These approaches rely on fusing the views into the shared latent space as the inference procedure, which often requires tailored training and testing paradigms when views are missing. However, adding information from multiple views may not lead to improved results and can even make the model perform worse on the targeted task.^12^^,^^13^ This is especially the case when views have noisy observations, which complicates learning a shared latent space that combines the commonalities between the views. To avoid disturbing the shared latent space with noise from single views, some works design models that extend the shared latent space with private latent spaces for each view that should contain the view-specific variations to make learning the shared variations easier.^3^^,^^57^^,^^60^^,^^65^^,^^72^ VCCA can be extended to extract shared and private information between different views through factorization of the latent space into shared and private parts. In this paper, we investigate whether the classification performance of grocery items in natural images can be improved by extracting the view-specific variations in the additional views (iconic images and product descriptions) from the shared latent space with this variant of VCCA, called VCCA-private. We experiment with treating each data point as pairs of natural images and either iconic images or text descriptions as well as triplets of natural images, iconic images, and text descriptions. A difference between how we apply VCCA to our dataset compared with the aforementioned works is that the iconic image and text description are the same for every natural image of a specific class.

## Results

In this section, we begin by providing the details about the collected dataset, which we have named the Grocery Store dataset. Furthermore, we illustrate the utility of the additional information in the Grocery Store dataset to classify grocery items in the experiments. We compare SplitAE, VCCA, and VCCA-private with different combinations of views against two standard image classifiers. Additionally, we experiment with a vanilla autoencoder (denoted as AE) and a VAE that post-processes the natural image features to train a linear classifier and compare the performance against the multi-view models. We measure the classification performance on the test set for every model and also compare the classification accuracies when the number of words in the text description varies for the models concerned (see Classification Results in Results). To gain insights into how the additional views affect the learned representations, we visualize the latent spaces of VCCA and VCCA-private with principal component analysis (PCA) and discuss how different views change the structure of the latent space (see Investigation of the Learned Representations in Results). Finally, we show how iconic images can be used for enhancing the interpretability of the classification (see Decoding Iconic Images from Unseen Natural Images in Results), which was also illustrated by Klasson et al.^2^

### The Grocery Store Dataset

**Figure 2:**
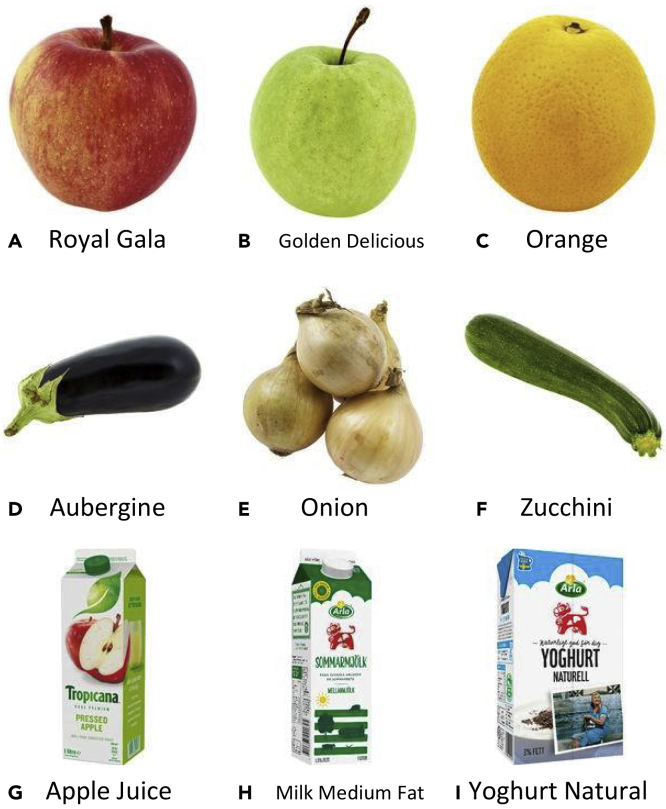
Examples of Iconic Images Downloaded from a Grocery Shopping Website, which Correspond to the Target Items in the Images in Figure 1

**Figure 3:**
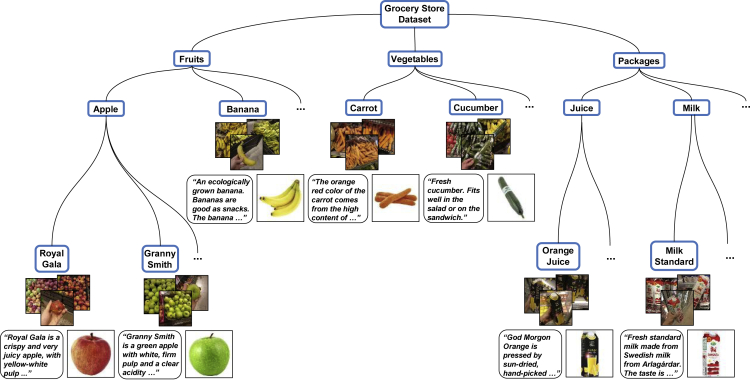
Illustration of the Hierarchical Class Structure of the Dataset First, the classes are divided by their grocery item type, i.e., Fruits, Vegetables, and Packages, followed by separating the items into coarse-grained classes, e.g., Apple, Carrot, and Milk, then into fine-grained classes. There are 81 fine-grained classes in total and 46 coarse-grained classes. Also shown is the iconic image and text description of the items next to the class label.

In Klasson et al.,^2^ we collected images from fruit, vegetable, and refrigerated sections with dairy and juice products in 20 different grocery stores. The dataset consists of 5,421 images from 81 different classes. For each class, we have downloaded an iconic image of the item, a text description, and information including country of origin, appreciated weight, and nutrient values of the item from a grocery store website. Some examples of natural images and downloaded iconic images can be seen in Figures 1 and 2, respectively. Furthermore, Table S1 displays a selection of text descriptions with their corresponding iconic image. The text description length varies between 6 and 91 words with an average of 36 words. We also structure the classes hierarchically with three levels as illustrated in Figure 3. The first level divides the items into the three categories Fruits, Vegetables, and Packages. The next level consists of 46 coarse-grained classes, which divides the items into groups containing groups of item types, e.g., Apple, Carrot, and Milk. The third level consists of the 81 fine-grained classes where the items are completely separated. Note that a coarse-grained class without any fine-grained classes in the dataset, e.g., Banana and Carrot, is considered as a fine-grained class in the classification experiments (see Classification Results in Results), where we report both fine-grained and coarse-grained classification performance of the models.

We aimed to collect the natural images under similar conditions as if they would be taken with an assistive mobile application. All images have been taken with a 16-megapixel Android smartphone camera from different distances and angles. Occasionally, the images include other items in the background or even items that have been misplaced on incorrect shelves along with the targeted item. The lighting conditions in the images can also vary depending on where the items are located in the store. Sometimes the images are taken while the photographer is holding the item in the hand. This is often the case for refrigerated products, since these containers are usually stacked compactly in the refrigerators. For these images, we have consciously varied the position of the object, such that the item is not always centered in the image or present in its entirety. We split the natural images into a training set and test set based on the application need. Since the images have been taken in several different stores at specific days and time stamps, parts of the data will have similar lighting conditions and backgrounds for each photo occasion. To remove any such biasing correlations, we assigned all images of a certain class taken at a certain store to either the training set, validation set, or test set. In the first version of the dataset,^2^ we balanced the class sizes to a large extent as possible in both the training and test set, which resulted in a training and test set containing 2,640 and 2,485 images, respectively. In this paper, we have extended the dataset with a validation set containing 296 images taken with the same smartphone camera as before. The validation set was collected from two different grocery stores than the ones in the first version to avoid the biasing correlations described above. Initially, we experimented with grabbing a validation set from the current training set and noticed that the trained classifiers performed exceptionally well on the validation set. However, because the classifiers generalized poorly to images from the test set that were taken in other stores, we decided to collect the validation set in two unseen stores to avoid the biases from the training set. Histograms representing the class distributions for the training, validation, and test splits are shown in Figure S1.

The scenario that we wanted to depict with the dataset was using a mobile device to classify natural images for visually impaired people while grocery shopping. The additional information such as the iconic images, text descriptions, and hierarchical structure of the class labels can be used to enhance the performance of the computer vision system. Since every class label is associated with a text description, the description itself can be part of the output for visually impaired persons, as they may not be able to read what is printed on a carton box or a label tag on a fruit bin in the store.

### Models

In this section, we briefly describe the models that we apply to grocery classification. The notation of the views that are available in the Grocery Store dataset are the following.

• *x*: Natural image encoded into image feature space with an off-the-shelf convolutional neural network
• *i*: Iconic image of the object class in the natural image
• *w*: Text description of the object class in the natural image
• *y*: Class label of the natural image

We mainly focus on analyzing VCCA^3^ for utilizing different combinations of the views and investigate how each view contributes to the classification performance of grocery items. Our primary baseline model is the SplitAE, which extracts shared representations by reconstructing all views, while VCCA aims to maximize a lower bound on the data log likelihood for all views. We also study a variant of VCCA called VCCA-private,^3^ which is used for extracting private information about each view in addition to shared information across all views by factorizing the latent space.

We compare the multi-view models against single-view methods that only use the natural images for classification. As our first single-view baseline, we customize the output layer of DenseNet169^73^ to our Grocery Store dataset and train it from scratch to classify the natural images, which we refer to as DenseNet-scratch in the experiments. The second baseline, called Softmax, is a Softmax classifier trained on the off-the-shelf features from DenseNet169 pre-trained on the ImageNet dataset,^15^ whereby we extract 1,664-dimensional from the average pooling layer before the classification layer in the architecture. We also experiment with AEs and VAEs for post-processing the off-the-shelf features and use a linear classifier to evaluate the models on classification. See Methods in Experimental Procedures for a thorough description of the single- and multi-view autoencoders used in this paper. We name the single- and multi-view autoencoders using subscripts to denote the views utilized for learning the shared latent representations. For example, VCCA_*xi*_ utilizes natural image features *x* and iconic images *i*, while VCCA_*xiwy*_ uses natural image features *x* and iconic images *i*, text descriptions *w*, and class labels *y*.

### Classification Results

We evaluated the classification accuracy on the test set for each model. We also calculated the accuracy for the coarse-grained classes using the following method. Let the input $x^{\left(i\right)}$ have a fine-grained class $y_{fg}^{\left(i\right)}$ and a coarse-grained class $y_{cg}^{\left(i\right)}$. Each fine-grained class can be mapped to its corresponding coarse-grained class using $Pa\left(y_{fg}^{\left(i\right)}\right)=y_{cg}^{\left(i\right)}$, where Pa(⋅) stands for “parent.” We then compute the coarse-grained accuracy using

(Equation 1) $$\begin{array}{l} \text{coarse accuracy}=\frac{1}{N}\sum_{i=1}^{N}\left[Pa\left({\hat{y}}_{fg}^{\left(i\right)}\right)=y_{cg}^{\left(i\right)}\right]\\ {\hat{y}}_{fg}^{\left(i\right)}=\underset{y}{\mathrm{arg max}}\;p\left(y|x^{\left(i\right)}\right), \end{array}$$

where $\left[Pa\left({\hat{y}}_{fg}^{\left(i\right)}\right)=y_{cg}^{\left(i\right)}\right]=1$ when the condition is true and ${\hat{y}}_{fg}^{\left(i\right)}$ is the predicted fine-grained class from the selected classifier. The classification results for all models are shown in Table 1. We group the results in the table according to the utilized views and classifier. We see that Softmax trained on off-the-shelf features outperforms DenseNet-scratch by 4%. This result is common when applying deep learning to small image datasets, whereby pre-trained deep networks transferred to a new dataset usually perform well compared with training neural networks on the dataset from scratch. Therefore, we present results using the off-the-shelf features for all other models.

**Table 1:** Classification Accuracies on the Test Set for All Models Given as Percentage for Each Model

Model	Accuracy (%)	Coarse Accuracy (%)
DenseNet-scratch	67.33 ± 1.35	75.67 ± 1.15
Softmax	71.67 ± 0.28	83.34 ± 0.32
AE_*x*_ + Softmax	70.69 ± 0.82	82.42 ± 0.58
VAE_*x*_ + Softmax	69.20 ± 0.46	81.24 ± 0.63
SplitAE_*xy*_	70.34 ± 0.56	82.11 ± 0.38
VCCA_*xy*_	70.72 ± 0.56	82.12 ± 0.61
SplitAE_*xi*_ + Softmax	77.68 ± 0.69	87.09 ± 0.53
VCCA_*xi*_ + Softmax	77.02 ± 0.51	86.46 ± 0.42
VCCA-private_*xi*_ + Softmax	73.04 ± 0.56	84.16 ± 0.51
SplitAE_*xiy*_	77.43 ± 0.80	87.14 ± 0.57
VCCA_*xiy*_	77.22 ± 0.55	86.54 ± 0.51
VCCA-private_*xiy*_	74.04 ± 0.83	84.59 ± 0.83
SplitAE_*xw*_ + Softmax	76.27 ± 0.66	86.45 ± 0.56
VCCA_*xw*_ + Softmax	75.37 ± 0.46	86.00 ± 0.32
VCCA-private_*xw*_ + Softmax	75.11 ± 0.81	85.91 ± 0.55
SplitAE_*xwy*_	75.78 ± 0.84	86.13 ± 0.63
VCCA_*xwy*_	74.72 ± 0.85	85.59 ± 0.78
VCCA-private_*xwy*_	74.92 ± 0.74	85.59 ± 0.67
SplitAE_*xiw*_ + Softmax	77.79 ± 0.48	87.12 ± 0.62
VCCA_*xiw*_ + Softmax	77.51 ± 0.51	86.69 ± 0.41
SplitAE_*xiwy*_	78.18 ± 0.53	87.26 ± 0.46
VCCA_*xiwy*_	77.78 ± 0.45	86.88 ± 0.47

The subscript letters in the model names indicate the data views used in the model. The column Accuracy corresponds to the fine-grained classification accuracy. The column Coarse Accuracy corresponds to classification of a class within the correct parent class. Results are averaged using 10 different random seeds reported as mean and standard deviation. AE, Autoencoder; VAE, Variational Autoencoder; SplitAE, Split Autoencoder; VCCA, Variational Canonical Correlation Analysis.

The SplitAE and VCCA models surpass the Softmax baseline in classification performance when incorporating either the iconic image or text description view. We believe that the models using the iconic images achieve better classification accuracy over models using the text description because the iconic images contain visual features, e.g., color and shape of items, that are more useful for the image classification task. The text descriptions include more often information about the flavor, origin, and cooking details rather than describing visual features of the item, which can be less informative when classifying items from images. In most cases, the corresponding SplitAE and VCCA models perform equally well for classification performance. However, VCCA-private with iconic images results in a significant drop in accuracy compared with its counterpart. We observed that the private latent variable simply models noise, since there is only a single iconic image (and text description) for each class. We provide a further explanation of this phenomenon in Investigation of the Learned Representations in Results.

**Figure 4:**
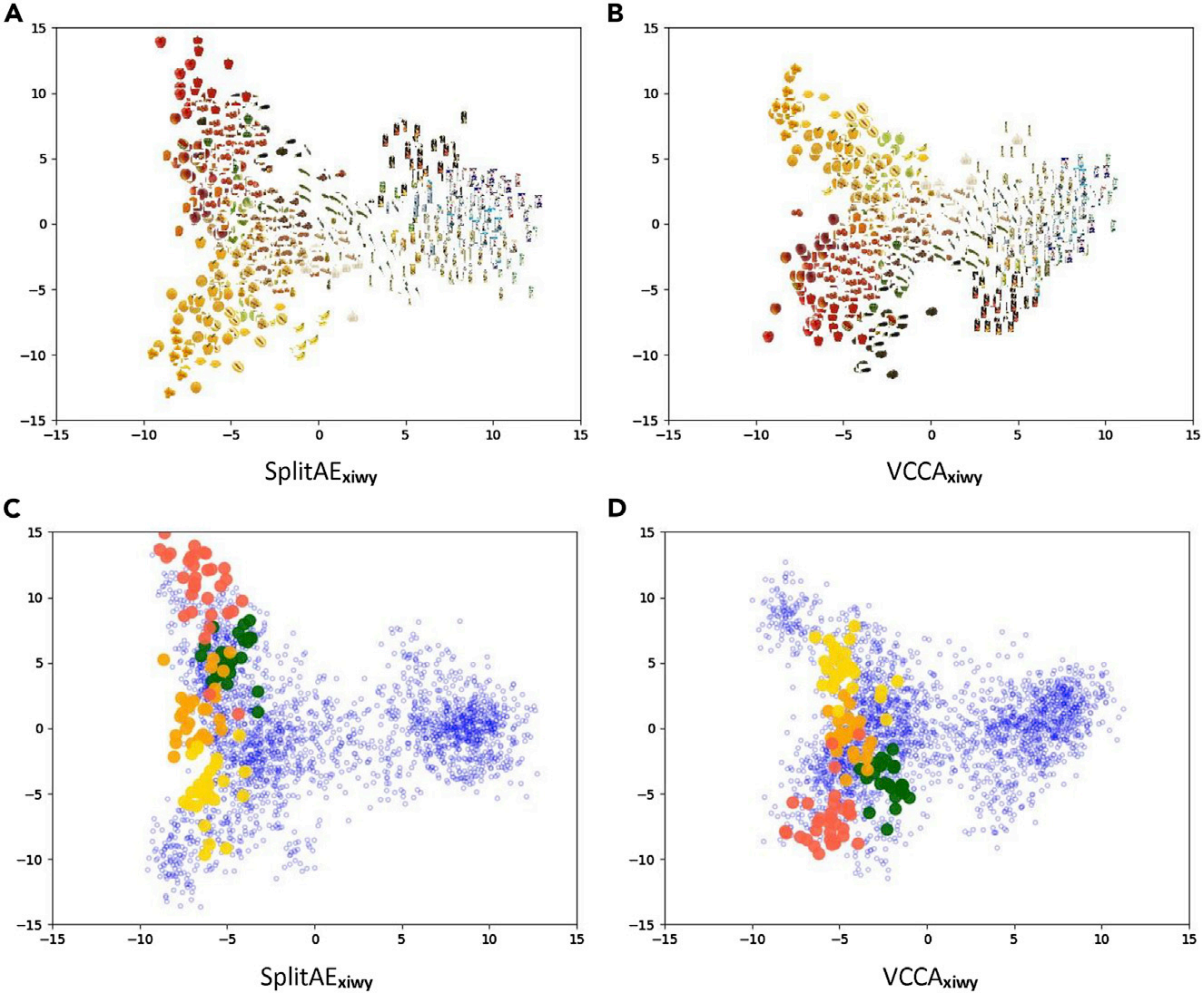
Visualizations of the Latent Representations of the Test Set from SplitAE_*xiwy*_ and VCCA_*xiwy*_ We plot the corresponding iconic image to each latent representation in (A) and (B). In (C) and (D), we plot the bell pepper representations according to the color of the class, while the blue points correspond to the other grocery items. SplitAE, Split Autoencoder; VCCA, Variational Canonical Correlation Analysis.

We observe that VCCA models compete with their corresponding SplitAE models in the classification task. The main difference between these models is the Kullback-Leibler (KL) divergence^74^ term in the evidence lower bound (ELBO) that encourages a smooth latent space for VCCA (see Experimental Procedures). In contrast, SplitAE learns a latent space that best reconstructs the input data, which can result in parts of the space that do not represent the observed data. We showcase these differences by plotting the latent representations of SplitAE_*xiwy*_ and VCCA_*xiwy*_ using PCA in Figure 4. In Figures 4A and 4B, we have plotted the corresponding iconic image for the latent representations. We observe that VCCA_*xiwy*_ tries to establish a smooth latent space by pushing visually similar items closer to each other but at the same time prevent spreading out the representations too far from the origin. Figures 4C and 4D shows the positions of the bell pepper items in the latent spaces, where the color of the point corresponds to the specific bell pepper class. In Figure 4C, we observe that SplitAE_*xiwy*_ has spread out the bell peppers across the space, while VCCA_*xiwy*_ establishes shorter distances between them in Figure 4D due to the regularization.

**Figure 5:**
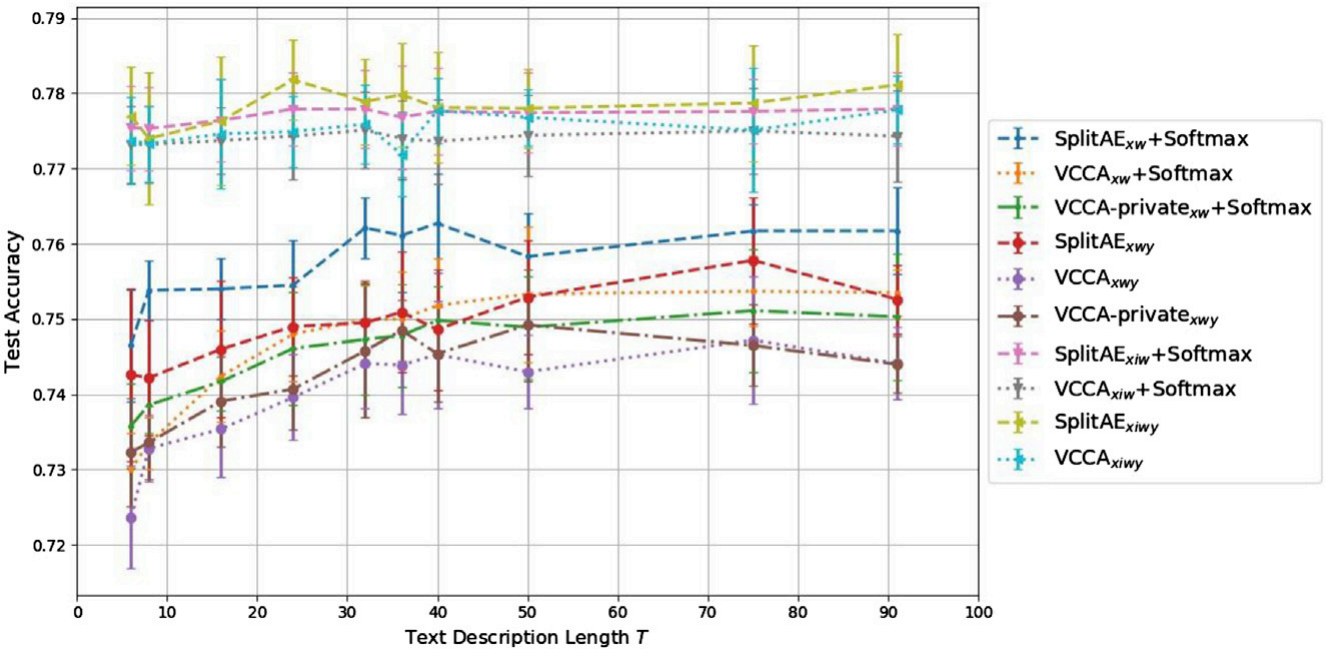
Test Accuracy over the Text Description Length *T* for all SplitAE, VCCA, and VCCA-Private Models Using the Text Description We show the accuracy for the models trained with *T* = 6, 8, 16, 24, 32, 36, 40, 50, 75, and 91 words. The results have been averaged for 10 different seeds and the error bars show the standard deviations for every setting of *T*. SplitAE, Split Autoencoder; VCCA, Variational Canonical Correlation Analysis.

We evaluated the classification performance achieved by each SplitAE, VCCA, and VCCA-private model using the text descriptions with different description lengths *T*. Figure 5 shows the fine-grained classification accuracies for the concerned models. For models using only the text descriptions, the classification accuracies increase as *T* increases in most cases. Setting T≥32 results in good classification performance, potentially since the models have learned to separate the grocery items based on that the text descriptions have become more dissimilar and unique. The classification accuracies are mostly stable as *T* varies for the models with the additional iconic images. Since including iconic images significantly increases the classification performance over models using only text descriptions, we conclude that the iconic images are more helpful when we want to classify the grocery items from natural images.

### Investigation of the Learned Representations

**Figure 6:**
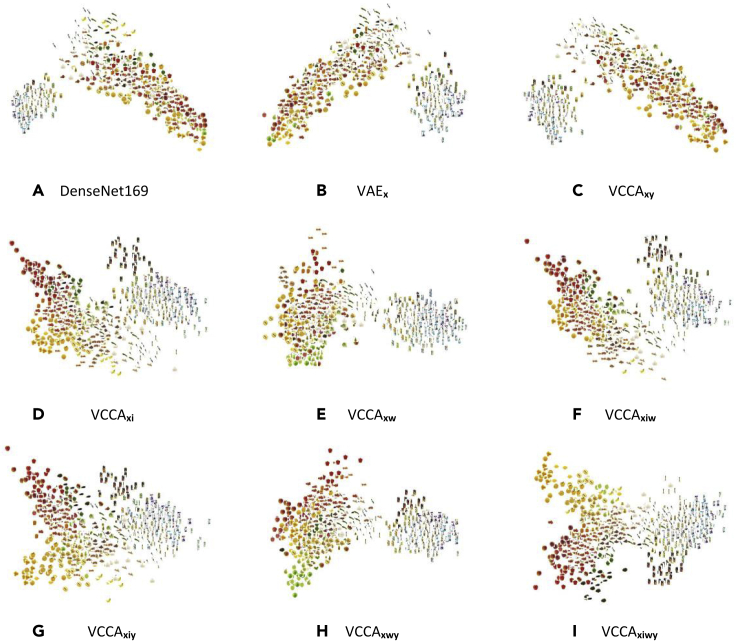
Visualizations of the Latent Representations from the Test Set, whereby We Plot the Iconic Image of the Corresponding Object Classes We also plot the PCA projection of the natural image features from the off-the-shelf DenseNet169 in (A). All models have been initialized with the same random seed before training. VAE, Variational Autoencoder; VCCA, Variational Canonical Correlation Analysis.

**Figure 7:**
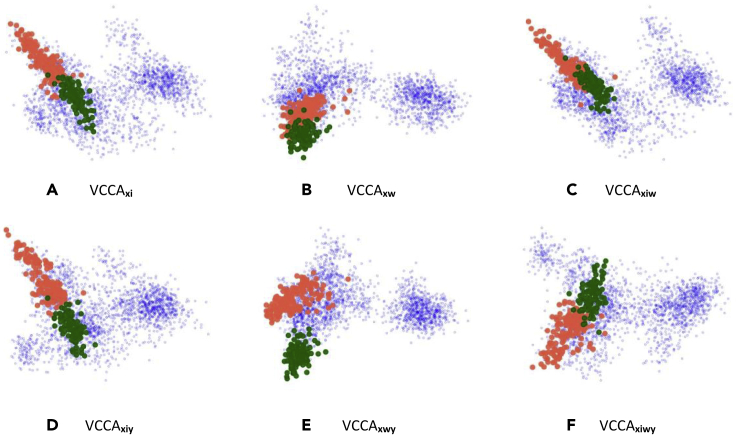
Visualizations of the Latent Representations *μ*_*z*_ of the Red and Green Apples in the Grocery Store Dataset The red points correspond to the red apple classes while the green points correspond to the green apple classes. The blue points correspond to the other grocery items. VCCA, Variational Canonical Correlation Analysis.

**Figure 8:**
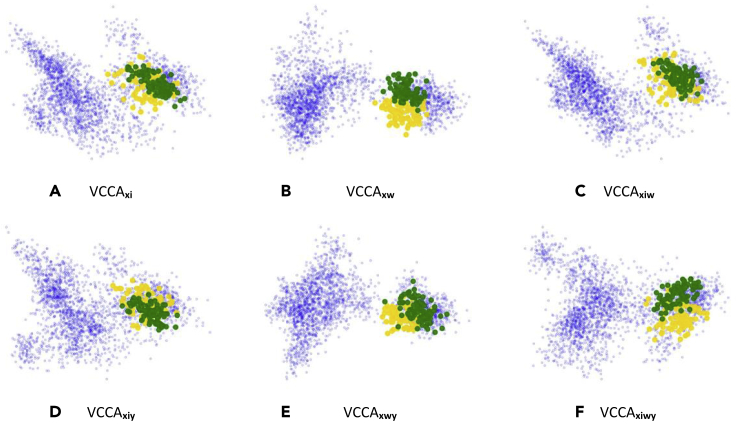
Visualizations of the Latent Representations *μ*_*z*_ of a Selection of Juice Packages and Yogurt Packages in the Grocery Store Dataset The yellow and green points correspond to the juice and yogurt packages, respectively. The blue points correspond to the other grocery items. VCCA, Variational Canonical Correlation Analysis.

To gain insights into the effects of each view on the classification performance, we visualize the latent space by plotting the latent representations using PCA. Utilizing the additional views showed similar effects on the structure of the latent spaces from SplitAE and VCCA. Since our main interest lies in representation learning with variational methods, we focus on studying the latent representations of VCCA and VCCA-private. First, we use PCA to visualize the latent representations in two dimensions and plot the corresponding iconic images of the representations (Figure 6). Second, to illustrate the effects of the iconic images and text descriptions on the learned latent space, we focus on two cases of grocery items whereby one of the views helps to separate two different types of classes and the other one does not (Figures 7 and 8). Finally, we look into the shared and private latent spaces learned by VCCA-private_*xw*_ and observe that variations in image backgrounds and structures of text sentences have been separated from the shared representations into the private ones.

In Figure 6, we show the latent representations for the VCCA models that were used in Table 1 (see Classification Results in Results). We also plot the PCA projections of the natural image features from the off-the-shelf DenseNet169 in Figure 6A as a baseline. Figures 6B and 6C show the latent space learned by *n* VAE_*x*_ and VCCA_*xy*_, which are similar to the DenseNet169 feature space since these models are performing compression of the natural image features into the learned latent space. We observe that these models have divided packages and raw food items into two separate clusters. However, the fruits and vegetables are scattered across their cluster and the packages have been grouped close to each other despite having different colors, e.g., black and white, on the cartons.

The structure of the latent spaces becomes distinctly different for the VCCA models that use either iconic images or text descriptions as an additional view, and we can observe the different structures that the views bring to the learned latent space. In Figures 6D and 6G, we see that visually similar objects, in terms of color and shape, have moved closer together by utilizing iconic images in VCCA_*xi*_ and VCCA_*xiy*_. When using text descriptions in VCCA_*xw*_ and VCCA_*xwy*_, we also observe in the fruit and vegetable cluster that the items are more grouped based on their color in Figures 6E and 6H. Figures 6F and 6I show the latent spaces in VCCA_*xiw*_ and VCCA_*xiwy*_, respectively. These latent spaces are similar to the ones learned by VCCA_*xi*_ and VCCA_*xiy*_ in the sense that these latent spaces also group items based on their color and shape. We believe that this structure imposed by the iconic images could be softened by reducing the scaling weight $\lambda_{i}$, which potentially could reduce the classification accuracy as a consequence. The difference between the latent spaces is not evident when comparing the models using the class label.

#### Red and Green Apples

To showcase how the iconic images help to learn good representations, we consider all of the apple classes in the dataset, namely the red apples Pink Lady, Red Delicious, and Royal Gala, and also the green apples Golden Delicious and Granny Smith. In Figure 7, we group the red apple classes and visualize their latent representations by red points. The green apples are grouped similarly and we visualize their latent representations with green points. Latent representations of all other grocery items are visualized as blue points. The models using iconic images as one view in Figures 7A, 7C, 7D, and 7F have managed to separate the red and green apples based on their color differences. The models using text description have instead moved the apples closer together in one part of the latent space, possibly because of their similarities mentioned in the description.

#### Juice and Yogurt Packages

To illustrate how the text descriptions can establish more useful latent representations, we consider a selection of juice and yogurt classes. These packaged items have similar shapes and colors, which makes it difficult for a classifier to distinguish their content differences using only visual input. In Figure 8, we visualize the latent representations of the juice and yogurt packages using yellow and green points, respectively. We observe that only VCCA_*xw*_ and VCCA_*xwy*_ manages to separate the packages in Figures 8B and 8E due to their different text descriptions. Since the iconic images of the packages are visually similar, adding this information is insufficient for separating these packages in the latent space. This indicates that we gain different benefits from the iconic images and text descriptions when it comes to classifying grocery items.

#### Latent Spaces of VCCA-Private

**Figure 9:**
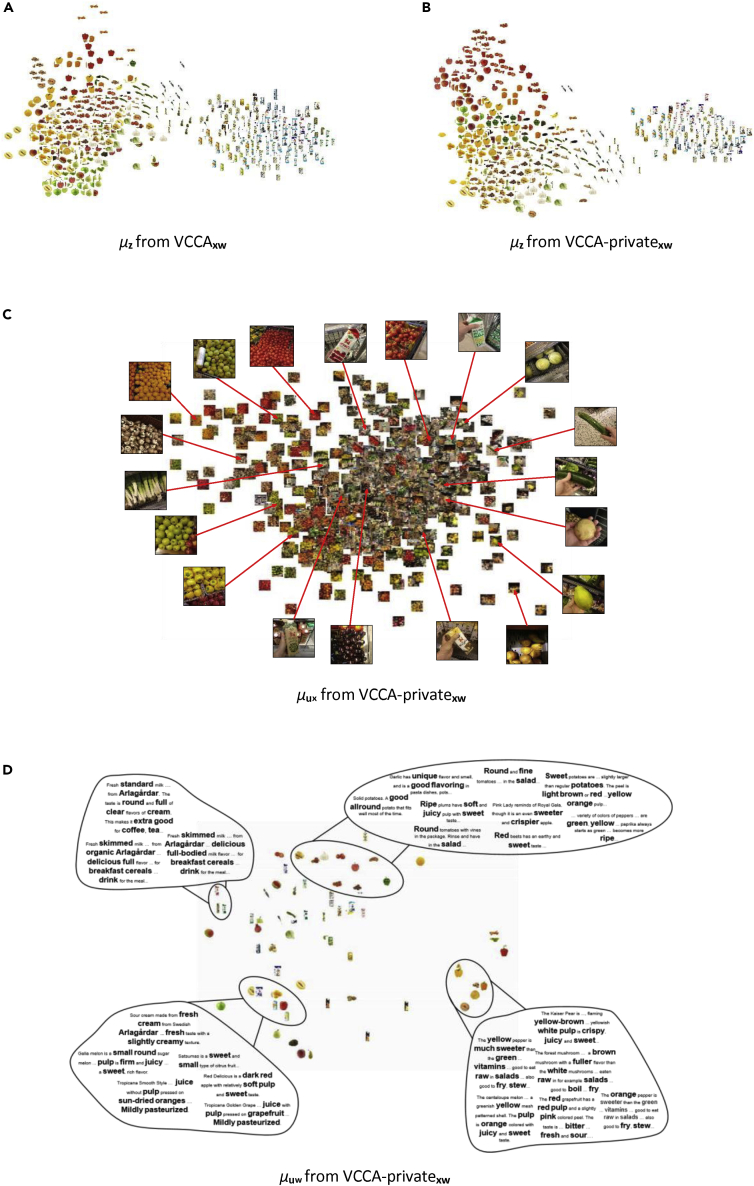
Visualizations of the Latent Representations *μ*_*z*_ from VCCA_*xw*_ and VCCA-private_*xw*_ Followed by *μ*_*uw*_ and *μ*_*ux*_ for VCCA-private_*xw*_ Representations from Variation Canonical Correlation Analysis (VCCA)_*xw*_ (A) and VCCA- private_*xw*_ (B), followed by *μ*_*uw*_ and *μ*_*ux*_ for VCCA-private_*xw*_ (C).

We show the shared and private latent spaces of VCCA-private_*xw*_ in Figures 9B–9D, as well as the single latent space of VCCA_*xw*_ for comparison in Figure 9A. Compared with the latent space of VCCA_*xw*_, the shared latent space of VCCA-private_*xw*_ in Figure 9B has structured the raw food items based on their class, color, and shape better than standard VCCA_*xw*_. In Figure 9C, we plot the natural images corresponding to the latent representation for the private latent variable $u_{x}$. We zoom in on some natural images and find that the images are structured based on their similarities in background and camera view. On the left and bottom sides of the cluster, we find images of grocery items closely packed together in bins. Single items that are held in the hand of the photographer are placed on the right side, whereas images of items and the store floor are placed on the top and middle of the latent space. The model has therefore managed to separate the variations within the natural image view into the private latent variable $u_{x}$ from the shared latent variable *z*, which probably is the main reason why similar raw food items are closer to each other in Figure 9B than in Figure 9A. We also plot the corresponding iconic image on the position of the text description representation for the private latent variable $u_{w}$ in Figure 9D. Note that every text description is projected at the same location in the latent space, since the text descriptions are the same for every class item. We highlighted some specific words in the descriptions and observed that descriptions with the same words are usually close to each other. Visually dissimilar items can be grouped close to each other in this latent space, which indicates that the private latent variable $u_{w}$ contains information about the structure of the text sentences, i.e., word occurrences and how they are ordered in the text description. In Figure S4, we show the shared and private latent spaces of VCCA-private_*xi*_, and provide a conclusion to the results in Supplemental Experimental Procedures.

### Decoding Iconic Images from Unseen Natural Images

In this section, we show how the iconic image decoder can decode plausible iconic images of grocery items from unseen natural images in the test set. We apply the same approach as in Klasson et al.,^2^ whereby we encode unseen natural images and then decode the retrieved latent representation back into an iconic image. We also report several image similarity metrics to investigate whether the decoded image quality is correlated with classification performance. We report peak signal-to-noise ratio (PSNR), structural similarity (SSIM),^75^ and the KL divergence by comparing the decoded iconic images with the true ones. For computing the KL divergence, we model the decoded and true iconic image using two Gaussian Mixture Models (GMMs).^76^^,^^77^ The images are then represented as density functions, such that we can measure the similarity between the two densities with the KL divergence and use it as an image similarity metric. Since the KL divergence between two GMMs is not analytically tractable, we apply Monte Carlo simulation using *n* independent and identically distributed samples drawn from the decoded image density for approximating the KL divergence.^78^ Due to the simple structure of the iconic images, we fit the GMMs with K=2 Gaussian components using the RGB color values and draw n=100 Monte Carlo samples to estimate the KL divergence in all experiments. Table 2 shows the image similarity metrics between the VCCA models using the iconic images. We also show the model classification accuracy for each model, which have been taken from Table 1. The models perform on par on the image similarity metrics, which indicates that the quality of the decoded images is intact if the model extends to utilizing text descriptions and class labels in addition to the iconic images.

**Table 2:** Results on Image Quality of Decoded Iconic Images for the Variational Canonical Correlation Analysis Models Using the Iconic Images

Model	PSNR (↑)	SSIM (↑)	KL (↓)	Accuracy (%)
VCCA_*xi*_	20.13 ± 0.05	0.72 ± 0.00	4.43 ± 0.21	77.02 ± 0.51
VCCA_*xiy*_	20.12 ± 0.09	0.73 ± 0.00	4.35 ± 0.22	77.22 ± 0.55
VCCA_*xiw*_	20.11 ± 0.09	0.73 ± 0.00	4.29 ± 0.24	77.51 ± 0.51
VCCA_*xiwy*_	20.16 ± 0.08	0.73 ± 0.00	4.32 ± 0.22	77.78 ± 0.45

The subscript letters in the model names indicate the data views used in the model. ↑ denotes higher is better, ↓ lower is better. Peak signal-to-noise ratio (PSNR), structural similarity (SSIM), and Kullback-Leibler divergence (KL) are measured by comparing the true iconic image against the decoded one. Accuracy shows the classification performance for each model and has been taken from Table 1. Data are reported as mean and standard deviation averaged over 10 random seeds for all metrics.

**Figure 10:**
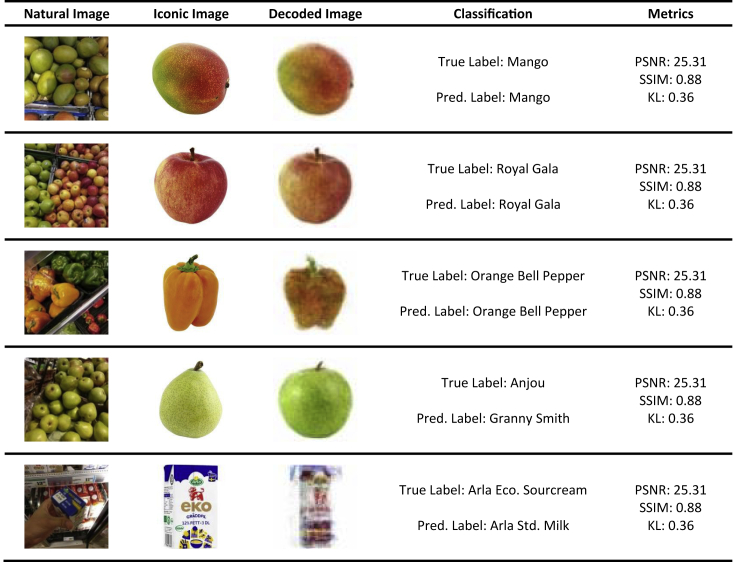
Examples of Decoded Iconic Images from VCCA_*xiwy*_ with Their Corresponding Natural Image and True Iconic Image as well as Predicted Labels and Image Similarity Metrics The column classification shows the true label for the natural image (True Label) and the label predicted by the model (Pred. Label). VCCA, Variational Canonical Correlation Analysis; PSNR, peak signal-to-noise ratio; SSIM, structural similarity; KL, Kullback-Leibler divergence; Arla Eco. Sourcream, Arla ecological sour cream; Arla Std. Milk, Arla standard milk.

In Figure 10, we display five different natural images from the test set, their true corresponding iconic image, and the decoded iconic image from VCCA_*xiwy*_. We also show the true and predicted labels from the class label decoder (Pred. Label). Additionally, we report the image similarity metrics PSNR, SSIM, and KL divergence between the decoded and true iconic images. For the Mango and Royal Gala images, we observe that the decoded images are visually plausible in terms of recognized item, color, and shape in both cases, which corresponds with the high PSNR and SSIM values and low KL values. The third row shows a shelf with orange and green bell peppers where the decoded image has indeed been decoded into a mix of a green and orange bell peppers. In the two succeeding rows, we display failure cases where the model confuses the true class label with other grocery items. We observe that each metric drops according to the mismatch between decoded and true iconic images. The fourth row shows a basket of Anjou pears, where the model confuses the pear with a Granny Smith apple which can be seen in the decoded image. In the fifth row, there are red milk packages stacked behind a handheld sour cream package, where the decoded image becomes a blurry mix of the milk and sour cream package. Although the predicted class is incorrect, we observe that the prediction is reasonable based on the decoded iconic image.

## Discussion

In this section, we summarize the experimental results and discuss our findings.

### Classification Results

In the first experiments (see Classification Results in Results), we showed that utilizing all four views with SplitAE_*xiwy*_ and VCCA_*xiwy*_ resulted in the best classification performance on the test set. This indicates that these models take advantage of each view to learn representations that enhance their generalization ability compared with the single-view models. Moreover, using the iconic images, the text descriptions, or both views yields better representations for classification compared with using the natural image features alone. Note that it was necessary to properly set the scaling weights *λ* on the reconstruction losses of the additional views to achieve good classification performance (see Table S2). Whenever the weight values are increased, the model tries to structure the representations according to variations between the items in the upweighted view rather than structuring the items based on visual features in the natural images, e.g., shape, color, and pose of items, and image backgrounds. Thus, the latent representations are structured based on semantic information that describes the item itself, which is important for constructing robust representations that generalize well in new environments. Furthermore, the class label decoders performed on par with the separate Softmax classifiers in most cases. The upside with training a separate classifier is that we only would have retrain the classifier if we receive a new class in the dataset, while we would have to train the whole model from scratch when the model uses a class label decoder. Note that the encoder for any of the multi-view models can be used for extracting latent representations for new tasks whether the model utilizes the label or not, since the encoder only uses natural images as input.

### Iconic Images versus Text Descriptions

In Table 1, the iconic images yielded higher classification accuracies compared with using the text descriptions. This was also evident in Figure 5 where the classification performance remains more or less the same regardless of the text description length *T* when the models utilize iconic images. We believe that the main reasons for the advantages with iconic images lie in the clear visual features of the items in these images, e.g., their color and shape, which carry much information that is important for image classification tasks. However, we also observed that iconic images and text descriptions can yield different benefits for constructing good representations. In Figure 6, we see that iconic images and text descriptions make the model construct different latent representations of the grocery items. Iconic images structure the representations with respect to color and shape of the items (Figure 7), while the descriptions group items based on their ingredients and flavor (Figure 8). Therefore, the latent representations benefit differently from utilizing the additional views and a combination of all of them yields the best classification performance, as shown in Table 1. We want to highlight the results in Figure 8, where the model manages to separate juice and yogurt packages based on their text description. Refrigerated items, e.g., milk and juice packages, have in general very similar shapes and the same color if they come from the same brand. There are minor visual differences between items of the same brand that makes it possible to differentiate between them, e.g., the picture of the main ingredient on the package and ingredient description. Additionally, these items can be almost identical depending on which side of the package that is present on the natural image. When utilizing the text descriptions, we add useful information on how to distinguish between visually similar items that have different ingredients and contents. This is highly important for using computer vision models to distinguish between packaged items without having to use other kinds of information, e.g., barcodes.

### Text Description Length

We showed that the text descriptions are useful for the classification task, and that careful selection of the description length *T* is important for achieving the best possible performance (see Classification Results in Results). In Figure 5, we observed that most models achieve significantly better classification performance when the text description length *T* increases up until T=32. The reason for this increase is due to the similarities between the descriptions of items from the same kind or brand, such as milk and juice packages. For instance, in Table S1, the first sentence in the descriptions for the milk packages only differs by the ninth word, which is “organic” for the ecological milk package. This means that their descriptions will be identical when T=8. Therefore, the descriptions will become more different from each other as we increase *T*, which helps the model to distinguish between items with similar descriptions. However, the classification accuracies have more or less saturated when setting T>32, which is also due to the similarity between the descriptions. For example, the bell pepper descriptions in Table S1 only differ by the second word that describes the color of the bell pepper, i.e., the words “yellow” and “orange.” We also see that the third and fourth sentences in the descriptions of the milk packages are identical. The text descriptions typically have words that separate the items in the first or second sentence, whereas the subsequent sentences provide general information on ingredients and how the item can be used in cooking. For items of the same kind but of different colors or brand, e.g., bell peppers or milk packages, respectively, the useful textual information for constructing good representations of grocery items typically comes from a few words in the description that describes features of the item. Therefore, the models yield better classification performance when *T* is set to include at least the whole first sentence of the description. We could select *T* more cleverly, e.g., by using different *T* for different descriptions to make sure that we utilize the words that describe the item or filter out noninformative words for the classification task.

### VCCA versus VCCA-Private

The main motivation for using VCCA-private is to use private latent variables for modeling view-specific variations, e.g., image backgrounds and writing styles of text descriptions. This could allow the model to build shared representations that more efficiently combine salient information shared between the views for training better classifiers. This would then remove noise from the shared representation, since the private latent variables are responsible for modeling the view-specific variations. For VCCA-private_*xw*_, we observed that the private latent spaces managed to group different image backgrounds and grocery items with similar text descriptions in Figures 9C and 9D, respectively. This model also performed on par with VCCA_*xw*_ regarding classification performance in Table 1. However, we also saw in the same table that the VCCA-private models using the iconic image perform poorly on the classification task compared with their VCCA counterpart. The reason why this model fails is because of a form of “posterior collapse”^79^ in the encoder for the iconic image, where the encoder starts outputting random noise. We noticed this as the KL divergence term for the private latent variable converged to zero when we trained the models for 500 epochs (see Figures S2 and S3), which means that the encoder outputs a distribution that equals a Gaussian prior. The same phenomenon also occurs for VCCA-private with the text description. We have also experimented with other models with encoders, such as Deep CCA and DCCAE, which also suffered from the collapsing encoder problem for the additional views. Therefore, we believe that the collapsing effect is a consequence of only having access to a single iconic image and text description for every grocery item. Therefore, a potential solution would be to extend the dataset with multiple web-scraped iconic images and text descriptions for every grocery item, which would then establish some variability within the view for each item class. Another possible solution would be to use data augmentation techniques to create some variability in the web-scraped views. For example, we could take a denoising approach and add noise to the iconic images, which would force the decoder to reconstruct the real iconic images.^80^ For the text descriptions, we could mask words at random in the encoder and let the decoder predict the whole description, which would work as a form of “word dropout.”^79^^,^^81^ We leave this for future work if such augmentation techniques can create the needed variability for learning more robust representations as well as discovering the structures of the private latent spaces that this approach would bring.

### Decoded Iconic Images

We observed with image similarity metrics that the quality of decoded iconic images coheres to some extent with the classification performance for VCCA models using the iconic images (see Decoding Iconic Images from Unseen Natural Images in Results). We also showed that the decoded images are visually plausible decoded images with respect to colors, shapes, and identities of the grocery items in the dataset. The values for the metrics PSNR, SSIM, and KL divergence are similar across the different VCCA models. Since we used RGB values for estimating KL divergence, we believe that including spatial information of pixels or using other color spaces, e.g., Lab, could provide more information about the dissimilarities between the decoded and true iconic images. To thoroughly assess the relationship between good classification performance and accurately decode the iconic images, we also suggest evaluating the image quality on other image similarity metrics, e.g., perceptual similarity.^82^ Finally, we see the decoding of iconic images as a promising method to evaluate the quality of the latent representations as well as enhancing the interpretability of the classification. For example, we could inspect decoded iconic images qualitatively or use image similarity metrics to determine how certain the model was about the present items in the natural images, which could then be used as a tool for explaining misclassifications.

### Conclusions

In this paper, we introduce a dataset with natural images of grocery items taken in real grocery store environments. Each item class is accompanied by web-scraped information in the form of an iconic image and a text description of the item. The main application for this dataset is for training image classifiers that can assist visually impaired people when shopping for groceries but is not limited to this utilization only.

We selected the multi-view generative model VCCA that can utilize all of the available data views for image classification. To evaluate the contribution to the classification performance for each view, we conducted an ablation study comparing classification accuracies between VCCA models with different combinations of the available data types. We showed that utilizing the additional views with VCCA yields higher accuracies on classifying grocery items compared with models only using the natural images. The iconic images and text descriptions impose different structures of the shared latent space, whereby we observed that iconic images help to group the items based on their color and shape while text descriptions separate the items based on differences in ingredients and flavor. These types of semantics that VCCA has learned can be useful for generalizing to new grocery items and other object recognition tasks. We also investigated VCCA-private, which introduces private latent variables for view-specific variations and separates the latent space into shared and private spaces for each view to provide high-quality representations. However, we observed that the private latent variables for the web-scraped views became uninformative by modeling noise due to the lack of variations in the additional web-scraped views. This encourages us to explore new methods for extracting salient information from such data views that can be beneficial for downstream tasks.

An evident direction of future work would be to investigate other methods for utilizing the web-scraped views more efficiently. For instance, we could apply pre-trained word representations for the text description, e.g., BERT^81^ or GloVe,^83^ to find out whether they enable the construction of representations that can more easily distinguish between visually similar items. Another interesting direction would be to experiment with various data augmentation techniques in the web-scraped views to create view-specific variations without the need for collecting and annotating more data. It is also important to investigate how the model can be extended to recognize multiple items. Finally, we see zero- and few-shot learning^63^ of new grocery items and transfer learning^84^ as potential applications for which our dataset can be used for benchmarking of multi-view learning models on classification tasks.

## Experimental Procedures

### Resource Availability

#### Lead Contact

Marcus Klasson is the lead contact for this study and can be contacted by email at mklas@kth.se.

#### Materials Availability

There are no physical materials associated with this study.

#### Data and Code Availability

1. The Grocery Store dataset along with documentation is available at the following Github repository: https://github.com/marcusklasson/GroceryStoreDataset
2. The source code for the multi-view models along with documentation is available at the following Github repository: https://github.com/marcusklasson/vcca_grocerystore

### Methods

In this section, we outline the details of the models we use for grocery classification. We begin by introducing autoencoders and SplitAEs.^14^ We then describe VAEs^70^ and how it is applied to single-view data, followed by the introduction of VCCA^3^ and how we adapt it to our dataset. We also discuss a variant of VCCA called VCCA-private,^3^ which is used for extracting private information about each view in addition to shared information across all views by factorizing the latent space. The graphical model representations of the VAE, VCCA, and VCCA-private models that have been used in this paper are shown in Figure S5. The model names use subscripts to denote the views utilized for learning the shared latent representations. For example, ${\text{VCCA}}_{xi}$ utilizes natural image features *x* and iconic images *i*, while ${\text{VCCA}}_{xiwy}$ uses natural image features *x* and iconic images *i*, text descriptions *w*, and class labels *y*.

#### Autoencoders and Split Autoencoders

The autoencoding framework can be used for feature extraction and learning latent representations of data in unsupervised manners.^85^ It begins with defining a parameterized function called the encoder for extracting features. We denote the encoder as $f_{\varphi }$ where *φ* includes its parameters, which commonly are the weights and bias vectors of a neural network. The encoder is used for computing a feature vector $h=f_{\varphi }\left(x\right)$ from the input data *x*. Another parameterized function $g_{\theta }$, called the decoder, is also defined, which maps the feature *h* back into input space, i.e., $\hat{x}=g_{\theta }\left(h\right)$. The encoder and decoder are learned simultaneously to minimize the reconstruction loss between the input and its reconstruction of all training samples. By setting the dimension of the feature vector smaller than the input dimension, i.e., $d_{h}<d_{x}$, the autoencoder can be used for dimensionality reduction, which makes the feature vectors suitable for training linear classifiers in a cheap way.

As in the case for the Grocery Store dataset, we have multiple views available during training, while only the natural image view is present at test time. In this setting, we can use a Split Autoencoder (SplitAE) to extract shared representations by reconstructing all views during training from the one view that is available during the test phase.^13^^,^^14^ As an example, we have the two-view case with *x* present at both training and test while *y* is only available during training. We therefore define an encoder $f_{\varphi }$ and two decoders $g_{\theta_{x}}$ and $g_{\theta_{y}}$_,_ where both decoders input the same representation $h=f_{\varphi }\left(x\right)$. The objective of the SplitAE is to minimize the sum of the reconstruction losses, which will encourage representations *h* that best reconstructs both views. The total loss is then

(Equation 2) $$L_{\mathrm{Split}\text{AE}}\left(\theta ,\varphi ;x,y\right)=\lambda_{x}L_{x}\left(x,g_{\theta_{x}}\left(h\right)\right)+\lambda_{y}L_{y}\left(y,g_{\theta_{y}}\left(h\right)\right),$$

where $\theta_{x},\theta_{y}\in \theta$ and $\lambda_{x},\lambda_{y}$ are scaling weights for the reconstruction losses. For images, the reconstruction loss can be the mean squared error, while the cross-entropy loss is commonly used for class labels and text. This architecture can be extended to more than two views by simply using view-specific decoders that input the shared representation extracted from natural images. Note that in the case when the class labels are available, we can use the class label decoder $g_{\theta_{y}}$ as a classifier during test time. Alternatively, we can train a separate classifier with the learned shared representations after the SplitAE has been trained.

#### Variational Autoencoders with Only Natural Images

The Variational Autoencoder (VAE) is a generative model that can be used for generating data from single views. Here, we describe how the VAE learns latent representations of the data and how the model can be used for classification. VAEs define a joint probability distribution $p_{\theta }\left(x,z\right)=p\left(z\right)p_{\theta }\left(x|z\right)$, where p(z) is a prior distribution over the latent variables *z* and $p_{\theta }\left(x|z\right)$ is the likelihood over the natural images *x* given *z*. The prior distribution is often assumed to be an isotropic Gaussian distribution, p(z)=N(z|0,I), with the zero vector **0** as mean and the identity matrix Ias the covariance. The likelihood $p_{\theta }\left(x|z\right)$ takes the latent variable *z* as input and outputs a distribution parameterized by a neural network with parameters *θ*, which is referred to as the decoder network. A common distribution for natural images is a multivariate Gaussian, $p_{\theta }\left(x|z)=N(x\;|\;\mu_{x}\left(z\right),\sigma_{x}^{2}\left(z\right)⊙I\right)$, where $\mu_{x}\left(z\right)$ and $\sigma_{x}^{2}\left(z\right)$ are the means and standard deviations of the pixels respectively outputted from the decoder and, ⊙ denotes element-wise multiplication. We wish to find latent variables *z* that are likely to have generated *x*, which is done by approximating the intractable posterior distribution $p_{\theta }\left(z|x\right)$ with a simpler distribution $q_{\varphi }\left(z|x\right)$.^71^ This approximate posterior $q_{\varphi }\left(z|x\right)$ is referred to as the encoder network, since it is parameterized by a neural network *φ*, which inputs *x* and outputs a latent variable *z*. Commonly, we let the approximate posterior to be Gaussian $q_{\varphi }\left(z|x)=N(z\;|\;\mu_{z}\left(x\right),\sigma_{z}^{2}\left(x\right)⊙I\right)$, where the mean $\mu_{z}\left(x\right)$ and variance $\sigma_{z}^{2}\left(x\right)$ are the outputs of the encoder. The latent variable *z* is then sampled using the mean and variance from the encoder with the reparameterization trick.^70^^,^^86^ The goal is to maximize a tractable lower bound on the marginal log likelihood of *x* using $q_{\varphi }\left(z|x\right)$:

(Equation 3) $$\text{log}p_{\theta }\left(x\right)\geq \;L\left(\theta ,\varphi ;x\right)=\;E_{q_{\varphi }\left(z|x\right)}\left[\;\text{log}p_{\theta }\left(x|z\right)\;\right]-D_{\text{KL}}\left(q_{\varphi }\left(z|x\right)\;||\;p\left(z\right)\right).$$

The last term is the KL divergence^74^ of the approximate posterior from the prior distribution, which can be computed analytically when $q_{\varphi }\left(z|x\right)$ and p(z) are Gaussians. The expectation can be viewed as a reconstruction loss term, which can be approximated using Monte Carlo sampling from $q_{\varphi }\left(z|x\right)$. The lower bound L is called the ELBO and can be optimized with stochastic gradient descent via backpropagation. The mean outputs $\mu_{z}\left(x\right)$ from the encoder $q_{\varphi }\left(z|x\right)$ are commonly used as the latent representation of the natural images *x*. We can use the representations $\mu_{z}\left(x\right)$ for training any classifier $p\left(y|\mu_{z}\left(x\right)\right)$, e.g., a Softmax classifier, where *y* is the class label of *x*. We can therefore see training the VAE as a pre-processing step, where we extract a lower-dimensional representation of the data *x* which hopefully makes the classification problem easier to solve. We can also extend the VAE with a generative classifier by incorporating the class label *y* in the model.^87^^,^^88^ Hence, the VAE defines a joint distribution $p_{\theta }\left(x,y,z\right)=p\left(z\right)p_{\theta_{x}}\left(x|z)p_{\theta_{y}}(y|z\right)$_,_ where the class label decoder $p_{\theta_{y}}\left(y|z\right)$ is used as the final classifier. We therefore aim to maximize the ELBO on the marginal log likelihood over *x* and *y*:

(Equation 4) $$\text{log}\;p_{\theta }\left(x,y\right)\geq \;L\left(\theta ,\varphi ;x,y\right)=\;\lambda_{x}E_{q_{\varphi }\left(z|x\right)}\left[\;\text{log}\;p_{\theta_{x}}\left(x|z\right)\right]+\lambda_{y}E_{q_{\varphi }\left(z|x\right)}\left[\;\text{log}\;p_{\theta_{y}}\left(y|z\right)\;\right]-D_{\text{KL}}\left(q_{\varphi }\left(z|x\right)\;||\;p\left(z\right)\right).$$

The parameters $\lambda_{x}$ and $\lambda_{y}$ are used for scaling the magnitudes of the expected values. When predicting the class label for an unseen natural image $x^{\text{∗}}$, we can consider multiple output predictions of the class label by sampling *K* different latent variables for $x^{\text{∗}}$ from the encoder to determine the final predicted class. For example, we could either average the predicted class scores over *K* or use the maximum class score from *K* samples as the final predictions. In this paper, we compute the average of the predicted class scores using

(Equation 5) $$\hat{y}=\underset{y}{\text{arg max}}\frac{1}{K}\sum_{k=1}^{K}p_{\theta_{y}}\left(y|z^{\left(k\right)}\right),\;z^{\left(k\right)}∼q_{\varphi }\left(z|x*\right),$$

where $\hat{y}$ is the predicted class for the natural image $x^{\text{∗}}$. We denote this model as VCCA_xy_ due to its closer resemblance to VCCA than VAE in this paper.

#### Variational Canonical Correlation Analysis for Utilizing Multi-View Data

In this section, we describe the details of Variational Canonical Correlation Analysis (VCCA)^3^ for our application. In the Grocery Store dataset, the views can be the natural images, iconic images, text descriptions, or class labels, and we can use any combination of those three in VCCA. To illustrate how we can employ this model to the Grocery Store dataset, we let the natural images *x* and the iconic images *i* be the two views. We assume that both views *x* and *i* have been generated from a single latent variable *z*. Similarly as with VAEs, VCCA defines a joint probability distribution $p_{\theta }\left(x,i,z\right)=p\left(z\right)p_{\theta_{x}}\left(x\;|\;z)p_{\theta_{i}}(i\;|\;z\right)$. There are now two likelihoods for each view modeled by the decoders $p_{\theta_{x}}\left(x\;|\;z\right)$ and $p_{\theta_{i}}\left(i\;|\;z\right)$ represented as neural networks with parameters $\theta_{x}$ and $\theta_{i}$. Since we want to classify natural images, the other available views in the dataset will be missing when we have received a new natural image. Therefore, the encoder $q_{\varphi }\left(z|x\right)$ only uses *x* as input to infer the latent variable *z* shared across all views, such that we do not have to use inference techniques that handle missing views. With this choice of approximate posterior, we receive the following ELBO on the marginal log likelihood over *x* and *i* that we aim to maximize:

(Equation 6) $$\text{log}\;p_{\theta }\left(x,i\right)\geq \;L\left(\theta ,\varphi ;x,i\right)=\;\lambda_{x}E_{q_{\varphi }\left(z|x\right)}\left[\;\text{log}\;p_{\theta_{x}}\left(x|z\right)\right]+\lambda_{i}E_{q_{\varphi }\left(z|x\right)}\left[\;\text{log}\;p_{\theta_{i}}\left(i|z\right)\;\right]-D_{\text{KL}}\left(q_{\varphi }\left(z|x\right)\;||\;p\left(z\right)\right).$$

The parameters $\lambda_{x}$ and $\lambda_{i}$ are used for scaling the magnitude of the expected values for each view. We provide a derivation of the ELBO for three or more views in Supplemental Experimental Procedures. The representations $\mu_{z}\left(x\right)$ from the encoder $q_{\varphi }\left(z|x\right)$ can be used for training a separate classifier. We can also add a class label decoder $p_{\theta_{y}}\left(y|z\right)$ to the model and use Equation 5 to predict the class of unseen natural images.

#### Extracting Private Information of Views with VCCA-Private

In the following section, we show how the VCCA model can be altered to extract shared information between the views as well as view-specific private information to enable more efficient posterior inference. Assuming that a shared latent variable *z* is sufficient for generating all different views may have its disadvantages. Since the information in the views is rarely fully independent or fully correlated, information only relevant to one of the views will be mixed with the shared information. This may complicate the inference of the latent variables, which potentially can harm the classification performance. To tackle this problem, previous works have proposed learning separate latent spaces for modeling shared and private information of the different views.^3^^,^^57^^,^^65^ The shared information should represent the correlations between the views while the private information represents the independent variations within each view. As an example, the shared information between natural and iconic images are the visual features of the grocery item, while their private information is considered as the various backgrounds that can appear in the natural images and the different locations of non-white pixels in the iconic images. For the text descriptions, the shared information would be words that describe visual features in the natural images, whereas the private information would be different writing styles for describing grocery items with text. We adapt the approach from Wang et al.^3^ called VCCA-private and introduce private latent variables for each view along with the shared latent variable. To illustrate how we employ this model in the Grocery Store dataset, we let the natural images *x* and the text descriptions *w* be the two views. The joint distribution of this model is written as

(Equation 7) $$p_{\theta }\left(x,w,z,u_{x},u_{w}\right)=p_{\theta_{x}}\left(x|z,u_{x})p_{\theta_{w}}(w|z,u_{w}\right)p\left(z\right)p\left(u_{x}\right)p\left(u_{w}\right),$$

where $u_{x}$ and $u_{w}$ are the private latent variables for *x* and *w*, respectively. To enable tractable inference of this model, we employ a factorized approximate posterior distribution of the form

(Equation 8) $$q_{\varphi }\left(z,u_{x},u_{w}|x,w)=q_{\varphi_{z}}(z|x\right)q_{\varphi_{x}}\left(u_{x}|x)q_{\varphi_{w}}(u_{w}|w\right),$$

where each factor is represented as an encoder network inferring its associated latent variable. With this approximate posterior, the ELBO for VCCA-private is given by

(Equation 9) $$\text{log}\;p_{\theta }\left(x,w\right)\geq L_{\text{private}}\left(\theta ,\varphi ;x,w\right)=\lambda_{x}E_{{q\varphi }_{z}\left(z|x),q_{\varphi_{x}}(u_{x}|x\right)}\left[\text{log}\;p_{\theta_{x}}(x|z,u_{x})]+\lambda_{w}E_{{q\varphi }_{z}(z|x),q_{\varphi_{w}}(u_{w}|w)}[\text{log}\;p_{\theta_{w}}(w|z,u_{w})\right]-D_{\text{KL}}\left(q_{\varphi_{z}}\left(z|x\right)||p\left(z\right)\right)-D_{\text{KL}}\left(q_{\varphi_{x}}\left(u_{x}|x\right)\;||\;p\left(u\right)\right)-D_{\text{KL}}\left(q_{\varphi_{w}}\left(u_{w}|w\right)||p\left(u\right)\right)$$

The expectations in $L_{\text{private}}\left(\theta ,\varphi ;x,w\right)$ in Equation 9 can be approximated using Monte Carlo sampling from $q_{\varphi }\left(z|x\right)$. The sampled latent variables are concatenated and then used as input to their corresponding decoder. We let the approximated posteriors over both shared and private latent variables to be multivariate Gaussian distributions and their prior distributions to be standard isotropic Gaussians N(0,I). The KL divergences in Equation 8 can then be computed analytically. Since only natural images are present during test time and because the shared latent variable *z* should contain information about similarities between the views, e.g., the object class, we use the encoder $q_{\varphi }\left(z|x\right)$ to extract latent representations $\mu_{z}\left(x\right)$ for training a separate classifier. As for the VAE and standard VCCA, we can also add a class label decoder $p_{\theta_{y}}\left(y|z\right)$ only conditioned on *z* to the model and use Equation 5 to predict the class of unseen natural images. We evaluated the classification performance of VCCA-private and compared it with the standard VCCA model only using a single shared latent variable (see Classification Results in Results).

### Experimental Setup

This section briefly describes the network architecture designs and the selection of hyperparameters for the models. See Supplemental Experimental Procedures for full details of the network architectures and hyperparameters that we use.

#### Processing of Natural Images

We use a DenseNet169^73^ as the backbone for processing the natural images, since this architecture showed good classification performance in Klasson et al.^2^ As our first baseline, we customize the output layer of DenseNet169 to our Grocery Store dataset and train it from scratch to classify the natural images. For the second baseline, we train a Softmax classifier on off-the-shelf features from DenseNet169 pre-trained on the ImageNet dataset,^15^ where we extract 1664-dimensional from the average pooling layer before the classification layer in the architecture. Using pre-trained networks as feature extractors for smaller datasets has previously been proved to be a successful approach for classification tasks,^89^ which makes it a suitable baseline for the Grocery Store dataset. We denoted the DenseNet169 trained from scratch and the Softmax classifier trained on off-the-shelf features as DenseNet-scratch and Softmax, respectively, in the Results section.

#### Network Architectures

We use the same architectures for SplitAE and VCCA for a fair comparison. We train the models using off-the-shelf features extracted from a pre-trained DenseNet169 for the natural images. No fine-tuning of the DenseNet backbone was used in the experiments, which we leave for future research. The image feature encoder and decoder consist of a single hidden layer, where the encoder outputs the latent representation and the decoder reconstructs the image feature. We use a DCGAN^90^ generator architecture for the iconic image decoder reconstructing the iconic images. The text description is predicted sequentially using an LSTM network.^91^ The label decoder uses a single hidden layer with 512 hidden units and Leaky ReLU activation. The latent space dimension is $d_{z}=200$ for all SplitAE and VCCA models in the experiments. In the VCCA-private models, the encoder and decoders have the same architectures as in the VCCA models. We use a reversed DCGAN generator as the iconic image encoder. The text description encoder is an LSTM. We obtain an embedding for the description by averaging all of the hidden states $h_{t}$ generated from the LSTM, i.e., $\frac{1}{T}\sum_{t=1}^{T}h_{t}$, and input it to a linear layer that outputs the latent representation. The dimensions of the private latent spaces are the same as the shared latent space dimension $d_{z}=200$.

#### Training Details

In all experiments, we train all models for 200 epochs using the Adam optimizer^92^ with initial learning rate $10^{-4}$and hyperparameters $\beta_{1}=0.9$ and $\beta_{2}=0.999$. We apply the sum-of-squares loss for the natural and iconic images and the categorical cross-entropy loss for text descriptions and class labels. The latent representations for the AE and SplitAE are the output from the encoder, while for the VAE, VCCA, and VCCA-private we instead use the mean outputs $\mu_{z}\left(x\right)$ from the encoder. In VCCA-private, we use the mean outputs $\mu_{u_{x}}\left(x\right)$ and $\mu_{u_{w}}\left(w\right)$ for visualizing the private latent representations (see Figure 9). The Softmax classifiers are trained for 100 epochs using with initial learning rate $10^{-4}$and hyperparameters $\beta_{1}=0.5$ and $\beta_{2}=0.999$. We run a hyperparameter search for the scaling weights *λ* for the reconstruction losses of each view (see Supplemental Experimental Procedures for more details).

#### Choice of Text Description Length *T*

We wanted to investigate how the classification performance is affected by the text description length *T* for the SplitAE and VCCA models using the text description *w*. Since the LSTM predicts the text description sequentially, the computational time increases as the text description length increases. We began by setting T=16 because of how the sentence length was set for the image captioning model in Lu et al.^8^ We then created an interval by taking steps with 8 words up to T=40 and included the minimum, mean, and maximum text description lengths, which are *T* = 6, 36, and 91, respectively. We added two additional points at T=50 and *T* = 75, since for most models there was a slight increase in classification performance when *T* was increased from 40 to 91.

#### Computational Time

The computational time for the SplitAE and VCCA models differs depending on which views that are being used. We measured the number of seconds per epoch (s/epoch) on an Nvidia GeForce RTX 2080 Ti, where an epoch consists of the 2,640 training samples. Running experiments with the text description view took around 0.4–2.7 s/epoch with text description length T∈[6,91]. Adding the iconic image view and training the models took around 4.5–6.2 s/epoch depending on the text description length.

#### Visualization Method

We use PCA to visualize the latent space by plotting the latent representations from the trained encoders. PCA is used for projecting the representations from dimension $d_{z}$ to a 2D space. We obtain the principal components for the representations with the natural images from the training set. In all figures, we plot the representations of test set images given the principal components obtained from the training images (see Investigation of the Learned Representations in Results). To distinguish more easily between the class labels of the images, we occasionally use the iconic images from the dataset and plot the corresponding iconic image of the representation on its location in the 2D space.

## Supplemental Information

Document S1. Supplemental Experimental Procedures, Figures S1–S5, and Tables S1 and S2

Data S1. Supplemental Article from Winter Conference on Applications of Computer Vision (WACV) 2019

Document S2. Article plus Supplemental Information
