# Supplementary material for: Using Variational Multi-view Learning for Classification of Grocery Items
Source: Patterns (N Y). 2020 Nov 13;1(8):100143. doi: 10.1016/j.patter.2020.100143 (PMC7691398; doi:10.1016/j.patter.2020.100143)
Supplement: Document S1. Supplemental Experimental Procedures, Figures S1–S5, and Tables S1 and S2 [file mmc1.pdf]

PATTER, Volume *1*

Supplemental Information

Using Variational Multi-view Learning for Classification of Grocery Items

Marcus Klasson, Cheng Zhang, and Hedvig Kjellström

Supplemental Information

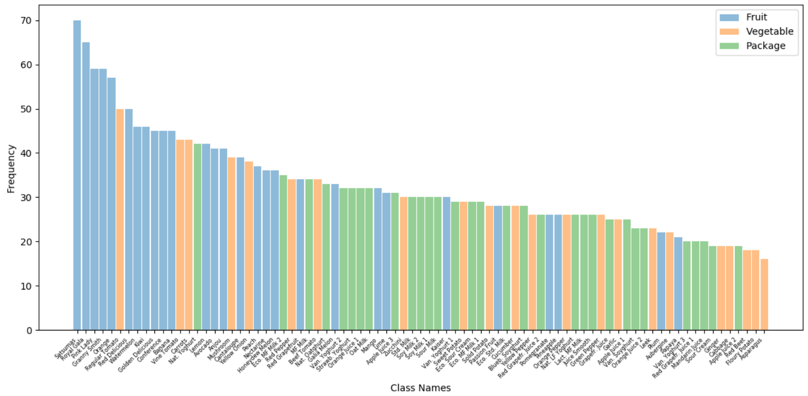

(a) Histogram for the training split.

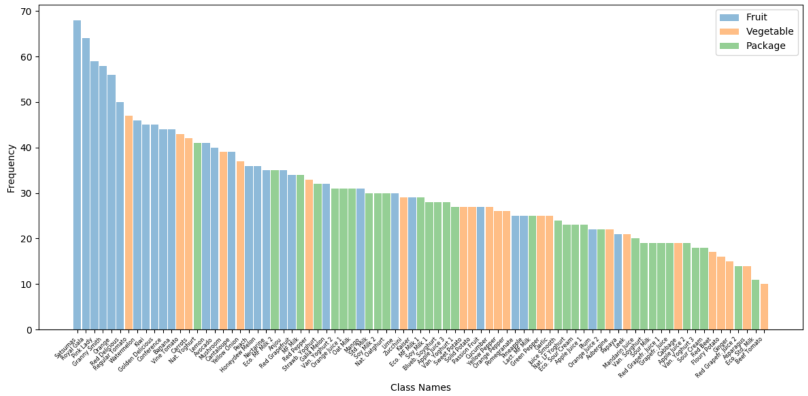

(b) Histogram for the test split.

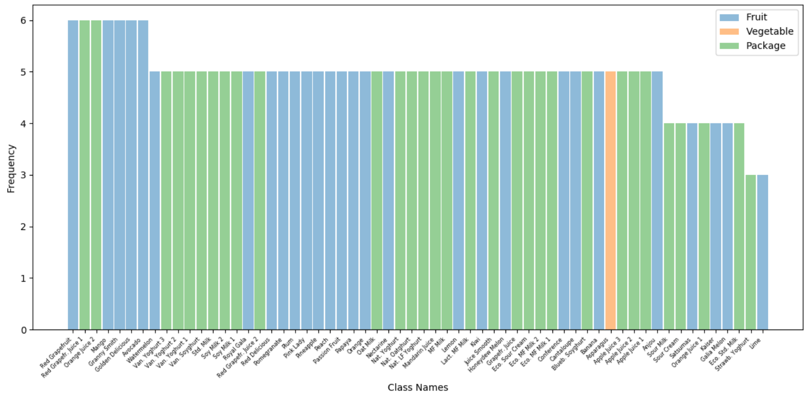

(c) Histogram for the validation split.

Figure S1: Histograms of natural images for every class in the training (a), test (b), and validation (c) splits of the Grocery Store dataset. We also show with different colors on the bins if the class is either a Fruit, Vegetable, or Package item.

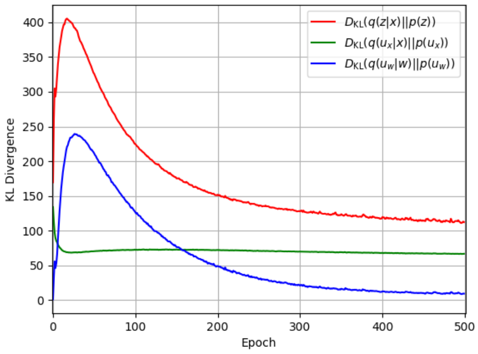

(a) VCCA-private$_{xw}$

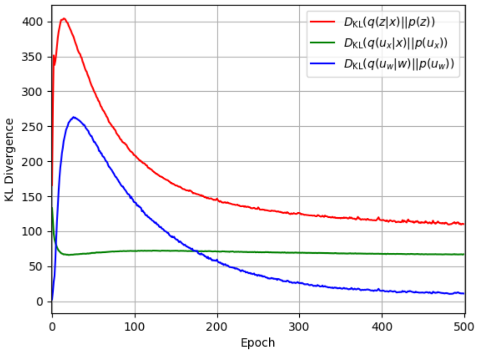

(b) VCCA-private$_{xwy}$

Figure S2: The measured KL divergences $D_{\mathrm{KL}}(q_{\phi_z}(z|x)\,||\,p(z))$ (red), $D_{\mathrm{KL}}(q_{\phi_x}(u_x|x)\,||\,p(u_x))$ (green), and $D_{\mathrm{KL}}(q_{\phi_w}(u_w|w)\,||\,p(u_w))$ (blue) over epochs. We increased the number of epochs from 200 to 500 to demonstrate that the KL divergence for the private latent variable u_w goes to zero. The number of words $T = 24$ is the same for both models. The likelihood weights are set to $\lambda_w = 1000$ and $\lambda_y = 100$. Abbreviations: VCCA, Variational Canonical Correlation Analysis; KL, Kullback-Leibler.

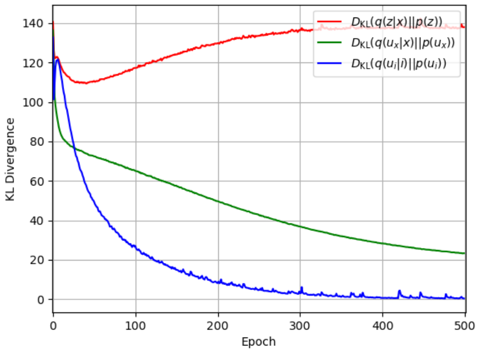

(a) VCCA-private$_{xi}$

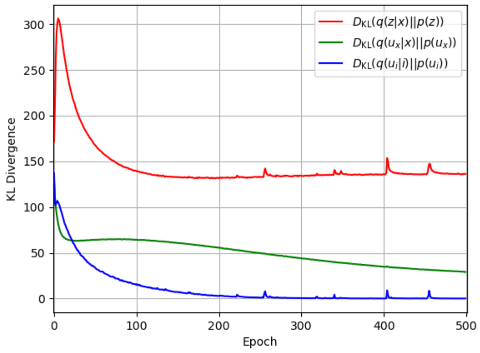

(b) VCCA-private$_{xiy}$

Figure S3: The measured KL divergences $D_{\mathrm{KL}}(q_{\phi_z}(z|x)\,||\,p(z))$ (red), $D_{\mathrm{KL}}(q_{\phi_x}(u_x|x)\,||\,p(u_x))$ (green), and $D_{\mathrm{KL}}(q_{\phi_i}(u_i|i)\,||\,p(u_i))$ (blue) over epochs. We increased the number of epochs from 200 to 500 to demonstrate that the KL divergence for the private latent variable u_i goes to zero. The likelihood weights are set to $\lambda_i = 10$ and $\lambda_y = 1000$. Abbreviations: VCCA, Variational Canonical Correlation Analysis; KL, Kullback-Leibler.

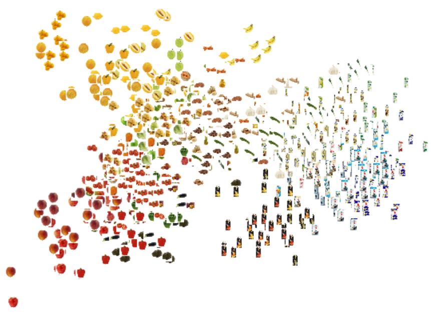

(a) μ_z from VCCA$_{xi}$

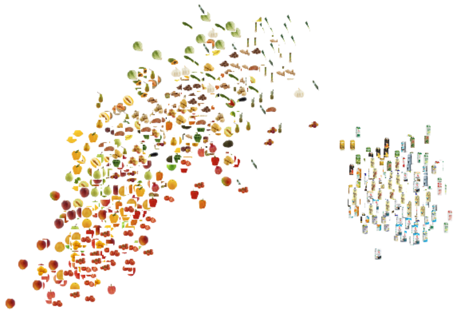

(b) μ_z from VCCA-private$_{xi}$

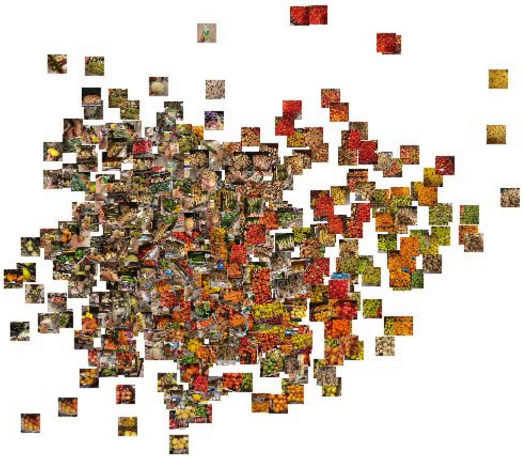

(c) μ_{u_x} from VCCA-private$_{xi}$

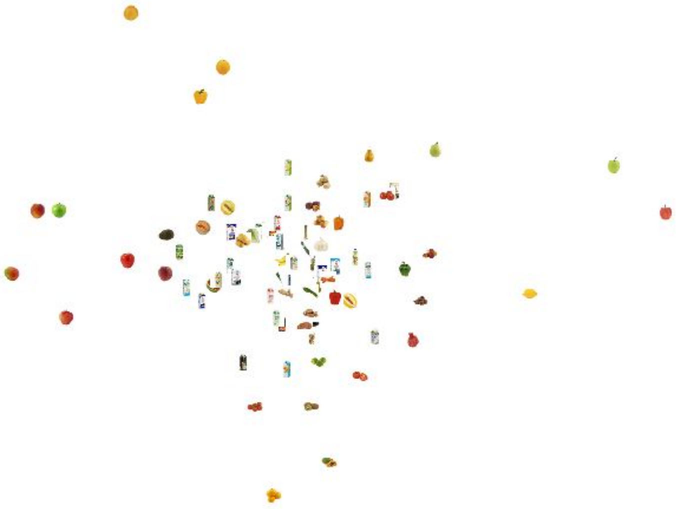

(d) μ_{u_i} from VCCA-private$_{xi}$

Figure S4: Visualizations of the latent representations $\mu_z(x)$ from VCCA$_{xi}$ and VCCA-private$_{xi}$ on the first row followed by $\mu_{u_x}(x)$ and $\mu_{u_i}(i)$ for VCCA-private$_{xi}$. Abbreviations: VCCA, Variational Canonical Correlation Analysis.

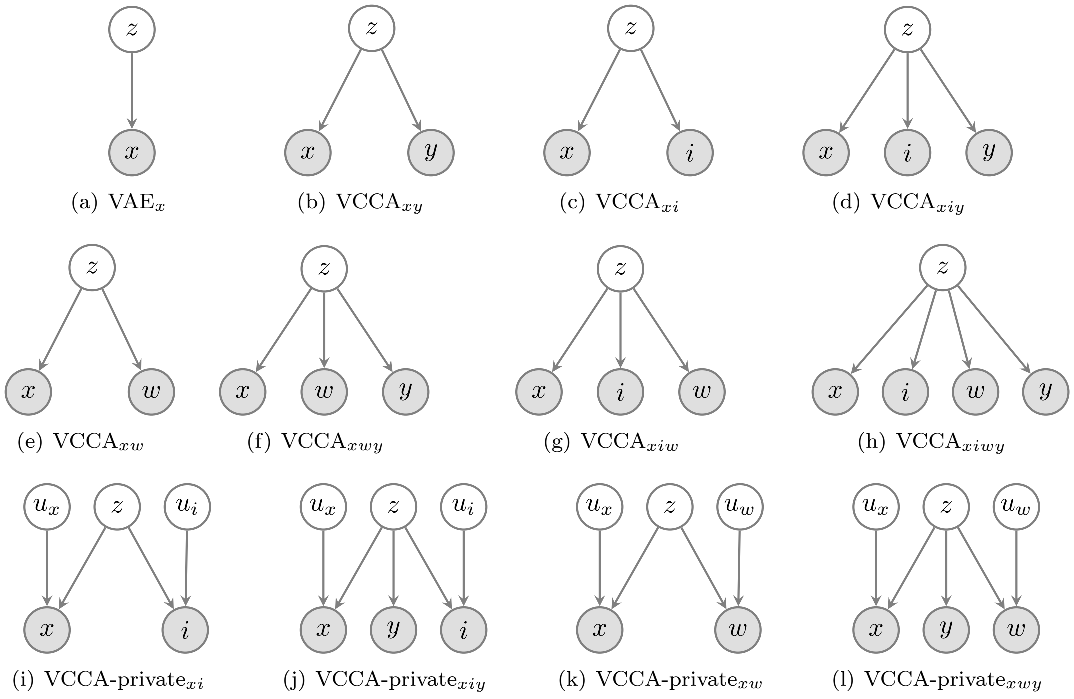

Figure S5: The graphical models of the VAE, VCCA, and VCCA-private, where nodes represent random variables and edges indicate possible dependence. Grey nodes are observed random variables, while white nodes are latent random variables. The joint distribution is given by the product of the conditional distributions of nodes given their parents. Abbreviations: VAE, Variational Autoencoder; VCCA, Variational Canonical Correlation Analysis.

Class Label	Iconic Image	Text Description
Golden Delicious Apple	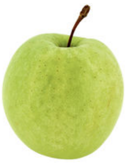	Golden Delicious has a white juicy pulp and a greenish yellow shell. The taste is mellow and sweet, making Golden Delicious suitable for desserts.
Red Delicious Apple	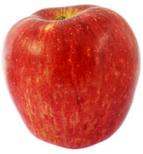	Red Delicious is a dark red apple with relatively soft pulp and sweet taste.
Orange	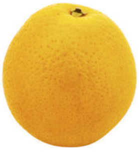	There are many different types of oranges that ripen and is sold during different parts of the year. The orange is a very important vitamin C source and the vitamins are best kept if the fruit is eaten naturally.
Yellow Bell Pepper	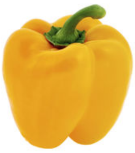	The yellow pepper is much sweeter than the green. It also contains more vitamins and antioxidants than the green. Peppers are good to eat raw in salads and as garnish, but are also good to fry, stew or gratinate, for example with filling. Paprika also fits well in pots, gratins and pies.
Orange Bell Pepper	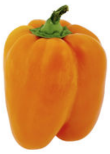	The orange pepper is sweeter than the green. It also contains more vitamins and antioxidants than the green. Peppers are good to eat raw in salads and as garnish, but are also good to fry, stew or gratinate, for example with filling. Paprika also fits well in pots, gratins and pies.
Arla Medium Fat Milk	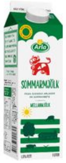	Fresh skimmed milk made from Swedish milk from Arlagårdar. Skimmed milk has a delicious full-bodied milk flavor and is a popular choice for breakfast cereals, porridge or as a drink for the meal. Milk is a natural source of, for example, protein, calcium and vitamin B12. Protein contributes to muscle building and calcium is needed to maintain a normal bone structure. The brand Arla Ko guarantees that the product is made of 100% Swedish milk.
Arla Ecological Medium Fat Milk	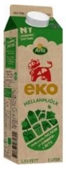	Fresh skimmed milk made from Swedish milk from organic Arlagårdar. Skimmed milk has a delicious full flavor and is a popular choice for breakfast cereals, porridge or as a drink for the meal. Milk is a natural source of, for example, protein, calcium and vitamin B12. Protein contributes to muscle building and calcium is needed to maintain a normal bone structure. The brand Arla Ko guarantees that the product is made of 100% Swedish milk. The new brown carton has 24 percent lower climate impact compared to the previous white carton.
God Morgon Orange Juice	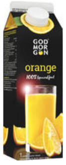	God Morgon Orange is pressed by sun-dried, hand-picked oranges. The package contains juice from 2 kilograms of oranges!
Tropicana Golden Grapefruit Juice	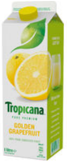	Tropicana Golden Grape is a ready to drink juice with pulp pressed on grapefruit. Not from concentrate. Mildly pasteurized.

Table S1: Examples of text descriptions and their corresponding class label and iconic image from the Grocery Store dataset.

Model	λ_x	λ_i	λ_w	λ_y	T	Accuracy (%)	Coarse Accuracy (%)
DenseNet-scratch	-	-	-	-	-	67.33 ± 1.35	75.67 ± 1.15
Softmax	-	-	-	-	-	71.67 ± 0.28	83.34 ± 0.32
AE_x+Softmax	1	-	-	-	-	70.69 ± 0.82	82.42 ± 0.58
VAE_x+Softmax	1	-	-	-	-	69.20 ± 0.46	81.24 ± 0.63
SplitAE_{xy}	1	-	-	1000	-	70.34 ± 0.56	82.11 ± 0.38
VCCA_{xy}	1	-	-	1000	-	70.72 ± 0.56	82.12 ± 0.61
SplitAE_{xi}+Softmax	1	1000	-	-	-	77.68 ± 0.69	87.09 ± 0.53
VCCA_{xi}+Softmax	1	1000	-	-	-	77.02 ± 0.51	86.46 ± 0.42
VCCA-private_{xi}+Softmax	1	10	-	-	-	73.04 ± 0.56	84.16 ± 0.51
SplitAE_{xiy}	1	1000	-	1000	-	77.43 ± 0.80	87.14 ± 0.57
VCCA_{xiy}	1	1000	-	1000	-	77.22 ± 0.55	86.54 ± 0.51
$\text{VCCA-private}_{xiy}$	1	10	-	1000	-	74.04 ± 0.83	84.59 ± 0.83
SplitAE_{xw}+Softmax	1	-	1000	-	40	76.27 ± 0.66	86.45 ± 0.56
VCCA_{xw}+Softmax	1	-	1000	-	75	75.37 ± 0.46	86.00 ± 0.32
VCCA-private_{xw}+Softmax	1	-	1000	-	75	75.11 ± 0.81	85.91 ± 0.55
SplitAE_{xwy}	1	-	1000	10	75	75.78 ± 0.84	86.13 ± 0.63
VCCA_{xwy}	1	-	1000	10	75	74.72 ± 0.85	85.59 ± 0.78
$\text{VCCA-private}_{xwy}$	1	-	1000	1000	50	74.92 ± 0.74	85.59 ± 0.67
SplitAE_{xiw}+Softmax	1	1000	1000	-	24	77.79 ± 0.48	87.12 ± 0.62
VCCA_{xiw}+Softmax	1	1000	1000	-	32	77.51 ± 0.51	86.69 ± 0.41
SplitAE_{xiwy}	1	1000	1000	1000	24	78.18 ± 0.53	87.26 ± 0.46
VCCA_{xiwy}	1	1000	1000	1000	91	77.78 ± 0.45	86.88 ± 0.47

Table S2: Classification accuracies on the test set for all models in percentage (%) along with the best hyperparameter setting, i.e., the scaling weights λ and text description length T, for each model. The column Accuracy corresponds to the fine-grained classification accuracy. The column Coarse Accuracy corresponds to the classifying a class within the correct parent class. Results are averaged using 10 different random seeds and we report both means and standard deviations. Abbreviations: AE, Autoencoder; VAE, Variational Autoencoder; SplitAE, Split Autoencoder; VCCA, Variational Canonical Correlation Analysis.

Supplemental Experimental Procedures

Details on Experimental Setup

In this section, we provide the full details on the experimental setups.

Training DenseNet169 from Scratch We train a DenseNet169 on the dataset from scratch as a baseline. We train the network using stochastic gradient descent for 300 epochs and follow the learning rate schedule of Huang *et al.*[1], i.e., using an initial learning rate of 0.1 and dividing it by 10 after 150 and 225 epochs. We use a weight decay of 10^{-4} and Nesterov momentum of 0.9 without dampening. We denote this network as DenseNet-scratch in the experiments.

Training the Softmax Classifier We use a Softmax classifier trained on off-the-shelf features as another baseline. We use a DenseNet169[1] pre-trained on ImageNet 1K as the feature extractor, where we extract 1664-dimensional from the average pooling layer before the classification layer in the architecture. The Softmax classifier is trained for 100 epochs with batch size 64 to minimize the cross-entropy loss. We use the Adam optimizer[2] with initial learning rate 10^{-4} and hyperparameters $\beta_1 = 0.5$ and $\beta_2 = 0.999$. Note that we used no regularization when training the Softmax classifier. We denote this classifier as Softmax in the experiments. This training setup is also used when training the Softmax classifiers for SplitAE, VCCA, and VCCA-private.

Architectures for Single-View Autoencoders We use a vanilla autoencoder and a VAE as baselines that learn latent representations of the natural image features only. These models are denoted as AE_x and VAE_x respectively in the experiments. Their latent representations have dimension $d_z = 200$ throughout all experiments. The encoder and decoder networks consist of one hidden layer of 512 hidden units with Leaky ReLU activation. The decoder aims to reconstruct the natural image features and we use the sum-of-squares as the reconstruction loss during training. For the VAE, we use the mean outputs $\mu_z(x)$ from the encoder as the latent representations of the image features to train a Softmax classifier, which we denote as VAE_x+Softmax.

Architectures for SplitAE and VCCA We use the same encoder and decoder architecture for the natural image features as in the single-view autoencoders for all SplitAE, VCCA, and VCCA-private models, i.e., one hidden layer of 512 hidden units with Leaky ReLU activation. We set the latent dimension to $d_z = 200$ in all experiments. The class label decoders use the same architecture as the image feature decoder and predict the class label by optimizing the cross-entropy loss. The iconic image decoders use the generator architecture of the DCGAN[3]. This decoder reconstructs the iconic images $i \in \mathbb{R}^{64\times64\times3}$ by minimizing the sum of squares loss. The text descriptions are assumed to be a sequence of words $w = (w_1, \dots, w_T)$, where T is the length of the description. We create a vocabulary $V \in \mathbb{R}^{658}$ of the total number of unique words from all text descriptions in the dataset. The text description decoder is an LSTM[4] followed by a linear layer that predicts the next word in the description. More specifically, at time step t, the decoder yields a multinomial probability distribution $p_{\theta_w}(w_t|w_{t-1}, z)$ defined over $w_t \in V$. The LSTM is trained using teacher forcing, meaning that we use the previous ground truth word as input at every time step during the training phase. We project each word into an embedding space of dimensionality $d_{emb} = 200$ by using a lookup table before the word is input to the LSTM. The hidden state h and memory state c of the LSTM is initialized with a linear projection of the latent representation z to provide the LSTM with some context about the natural image. We use the same dimension for h and c as for z, i.e., $d_z = d_h = d_c = 200$. We minimize the cross-entropy loss at every time step by comparing the predicted word with the true word in the description. We apply dropout[5] with a keep rate of 0.5 on the output hidden state h_t before we input it through the linear layer that predicts the word at time step t.

Architectures for VCCA-private In the VCCA-private models, the decoders has the same architectures as the VCCA models. The encoder for the shared latent variable z is also the same as the encoder for the previous described models. The encoder for the private latent variable u_x uses an identical architecture as encoder for z. We use a convolutional encoder for the iconic image private latent variable, which is a reversed DCGAN generator outputting the mean $\mu_{u_i}(i)$ and variance $\sigma^2_{u_i}(i)$. The text description encoder is an LSTM with the same architectural details as the

LSTM decoder. We obtain an embedding for the description by averaging all of the hidden states h_t generated from the LSTM, i.e., $\frac{1}{T}\sum_{t=1}^{T} h_t$, and input it to a linear layer outputting the mean $\mu_{u_w}(w)$ and the variance $\sigma^2_{u_w}(w)$ for the private latent variable for the text description. We use the same latent dimensions for the shared and private latent variables, i.e., $d_z = d_{u_x} = d_{u_i} = d_{u_w} = 200$.

Training the Single- and Multi-View Autoencoders The single- and multi-view autoencoding models are trained for 200 epochs with batch size 64 and aims to minimize either their reconstruction losses or their corresponding ELBOs. We use the Adam optimizer with initial learning rate 10^{-4} and hyperparameters $\beta_1 = 0.9$ and $\beta_2 = 0.999$ in all experiments. The mean outputs $\mu_z(x)$ from the encoder are used as the latent representations of the image features to train a Softmax classifier. For the class label decoder, we use $K = 1$ posterior samples for predicting the class label during training, while we set $K = 5$ in the validation and test stages.

Grid Search We run a hyperparameter search for the scaling weights λ for the reconstruction losses of the views using a grid search with grid points $\{0.1, 1, 10, 100, 1000\}$. The grid search is performed for all VCCA and VCCA-private models. The weight for the natural image feature loss λ_x is used as a reference by setting it to $\lambda_x = 1$ throughout all experiments. This means that we only vary the scaling weights λ_i, λ_w, and λ_y. We run the grid searches using three different random seeds and average the resulting validation accuracies to select the best hyperparameter setting. Table S2 shows the best hyperparameter settings for the VCCA and VCCA-private models with their validation accuracies. Note that we use the same scaling weights for SplitAE as the ones we found for its corresponding VCCA model. To summarize the grid search results, it is always beneficial to use scaling weights $\lambda > 1$ for the models to enhance the classification accuracy. This will make the models add some semantically meaningful information to the latent space from the additional views, which makes the models more suitable for downstream tasks such as classification.

Posterior Collapse in VCCA-private

We noticed in Table 1 that VCCA-private achieves similar classification performance as standard VCCA when utilizing the text description w and even worse results when utilizing the iconic image i. The private latent variables cannot capture any view-specific variations when each class uses the same iconic image and text description for every natural image. A consequence of this is that the iconic image and text description can be identified by only using the shared latent variable z in the decoding phase. The private latent variables are thus not necessary for determining which iconic image or text description to generate, the generated view will be the same anyway for every natural image of a specific class. The model then finds out that the ELBO can be maximized by letting the approximate posteriors be equal to their prior distributions, which minimize the KL divergences of the private latent variables. This phenomenon is referred to as *posterior collapse*[6].

In Figure S2 and S3, we illustrate that the approximate posterior deduces to its prior distribution – a zero-mean Gaussian with unit variance – during training. Figure S2(a) and S2(b) shows the KL divergences over epochs for VCCA-private$_{xw}$ and VCCA-private$_{xwy}$ respectively. The number of words $T = 24$ is the same for both models and we train the models for 500 epochs to emphasize that $D_{\mathrm{KL}}(q_{\phi_w}(u_w|w)\,||\,p(u_w))$ goes towards zero. We believe that this is due to that there are no variations in the text descriptions within one class (see subsection Investigations of the Learned Representations in Results). We perform a similar experiment with VCCA-private$_{xi}$ and VCCA-private$_{xiy}$ and plot their KL divergences in Figure S3. The KL divergence $D_{\mathrm{KL}}(q_{\phi_i}(u_i|w)\,||\,p(u_i))$ decreases to zero faster for these models than when we use the text description. We also observe that the KL divergence $D_{\mathrm{KL}}(q_{\phi_x}(u_x|x)\,||\,p(u_x))$ decreases to zero as well for both models. Why the KL divergence for the private latent variables decreases faster in the models with the iconic image i is mainly because their likelihood weight λ_i is smaller than λ_w. We noticed that the KL divergences of the private latent variables decreases slower towards zero when λ_i is increased, probably because the model foremost focuses on minimizing the reconstruction loss.

Investigating Latent Representations in VCCA-private$_{xi}$

Figure S4 shows the shared and private latent spaces of VCCA-private$_{xi}$, as well as the latent space of the standard VCCA$_{xi}$ for comparison. Note that the shared latent spaces in Figure S4(a) and S4(b) have different structures mainly due to the different settings of their likelihood

weight λ_i, which is $\lambda_i = 1000$ for VCCA$_{xi}$ and $\lambda_i = 10$ for VCCA-private$_{xi}$. We observe in Figure S4(c) that the natural images are structured based on their similarities in background and camera setup. Across the upper right and the lower left parts of the cluster, we find images of grocery items closely packed together in bins. The middle and upper left part includes images with the hand of the photographer and grey backgrounds, e.g., the floor and shelves in the grocery store. Note that this private latent space is rather densely packed mainly because the KL divergence $D_{\mathrm{KL}}(q_{\phi_x}(u_x|x)\,||\,p(u_x))$ is steadily decreasing towards zero (see Figure S3(a)). This means that the approximate posterior $q_{\phi_x}(u_x|x)$ is collapsing to its prior distribution, which is why the mean values μ_{u_x} are close to the origin of the space. The mean values μ_{u_i} for the private latent variable u_i are shown in Figure S4(d) using the iconic image. Note that every iconic image i is projected at the same location in the latent space. We observe that similar iconic images are projected close to each other. For instance, an orange, grapefruit and yellow bell pepper have been projected in the upper part, packaged items are in the center parts and the left region we find round objects with a dark red color. To the far right, we see a green and a red apple that has been pushed far away from the similar iconic images on the left. If we look closer into these iconic images, we observe that the apples on the right have a higher stalk on top of the apple than the apples on the left side has, which could be the reason why the model has separated them in the latent space. Another interesting observation is that iconic images with multiple items, e.g., tomatoes, kiwis and satsumas, have been projected into the lower region of the space. We conclude that similar iconic images, based on color and their appearance in the image, are grouped closely in the private latent space.

Derivation of the ELBO for VCCA

Let $x_{1:M}$ denote the all observed data, i.e., $x_{1:M} = x_1, \ldots, x_M$, for M different views. We derive the ELBO for VCCA by introducing the approximate posterior $q_\phi(z|x_m)$, where x_m is the only view that we use to infer the latent variable z. We now derive the ELBO from the marginal log-likelihood $\log p_\theta(x_{1:M})$ as

$$\begin{aligned}
\log p_\theta(x_{1:M}) &= \log p_\theta(x_{1:M}) \int q_\phi(z|x_m)\,dz \\
&= \int q_\phi(z|x_m) \log p_\theta(x_{1:M})\,dz \\
&= \int q_\phi(z|x_m) \log \frac{p_\theta(x_{1:M}, z)}{p_\theta(z|x_{1:M})}\,dz \\
&= \int q_\phi(z|x_m) \log \frac{p_\theta(x_{1:M}, z)}{p_\theta(z|x_{1:M})} \frac{q_\phi(z|x_m)}{q_\phi(z|x_m)}\,dz \\
&= \int q_\phi(z|x_m) \left(\log \frac{q_\phi(z|x_m)}{p_\theta(z|x_{1:M})} + \log \frac{p_\theta(x_{1:M}, z)}{q_\phi(z|x_m)} \right) dz \\
&= D_{\mathrm{KL}}(q_\phi(z|x_m)\,||\,p_\theta(z|x_{1:M})) + \mathbb{E}_{q_\phi(z|x_m)} \left[\log \frac{p_\theta(x_{1:M}, z)}{q_\phi(z|x_m)} \right] \\
&\geq \mathbb{E}_{q_\phi(z|x_m)} \left[\log \frac{p_\theta(x_{1:M}, z)}{q_\phi(z|x_m)} \right] = \mathcal{L}(x_{1:M}; \theta, \phi)
\end{aligned}$$

We use the factorization property of the joint distribution $p_\theta(x_{1:M})$ to further derive the ELBO $\mathcal{L}(x_{1:M}; \theta, \phi)$:

$$\begin{aligned}
\mathcal{L}(\theta, \phi; x_{1:M}) =& \mathbb{E}_{q_\phi(z|x_m)} \left[\log \frac{p_{\theta_1}(x_1|z) \cdots p_{\theta_M}(x_M|z) p(z)}{q_\phi(z|x_m)} \right] \\
=& \mathbb{E}_{q_\phi(z|x_m)} \left[\log p_{\theta_1}(x_1|z) + \ldots + \log p_{\theta_M}(x_M|z) + \log \frac{p(z)}{q_\phi(z|x_m)} \right] \\
=& \mathbb{E}_{q_\phi(z|x_m)} \left[\log p_{\theta_1}(x_1|z) \right] + \ldots + \mathbb{E}_{q_\phi(z|x_m)} \left[\log p_{\theta_M}(x_M|z) \right] - D_{\mathrm{KL}}(q_\phi(z|x_m)\,||\,p(z))
\end{aligned}$$

It may be necessary to balance the terms in the ELBO with some constant for every term, especially when the dimensions and magnitudes differ between the modalities. Thus, we introduce the likelihood weights $\lambda_1, \ldots, \lambda_M$, such that the ELBO will be written as

$$\begin{aligned}
\mathcal{L}(\theta, \phi; x_{1:M}) =& \lambda_1 \mathbb{E}_{q_\phi(z|x_m)} \left[\log p_{\theta_1}(x_1|z) \right] + \ldots + \lambda_M \mathbb{E}_{q_\phi(z|x_m)} \left[\log p_{\theta_M}(x_M|z) \right] \\
& - D_{\mathrm{KL}}(q_\phi(z|x_m)\,||\,p(z))
\end{aligned}$$

The likelihood weights $\lambda_1, ..., \lambda_M$ that are optimal for the task at hand can be found with some hyperparameter search.

References

[1] Huang, G., Liu, Z., Van Der Maaten, L., and Weinberger, K. Q., (2017). Densely connected convolutional networks. In *IEEE Conference on Computer Vision and Pattern Recognition.*

[2] Kingma, D. P. and Ba, J., (2015). Adam: A method for stochastic optimization. In *International Conference on Learning Representations.*

[3] Radford, A., Metz, L., and Chintala, S., (2015). Unsupervised representation learning with deep convolutional generative adversarial networks. *arXiv preprint arXiv:1511.06434.*

[4] Hochreiter, S. and Schmidhuber, J., (1997). Long short-term memory. *Neural computation*, 9(8): 1735–1780.

[5] Srivastava, N., Hinton, G., Krizhevsky, A., Sutskever, I., and Salakhutdinov, R., (2014). Dropout: A simple way to prevent neural networks from overfitting. *Journal of Machine Learning Research*, 15: 1929–1958.

[6] Bowman, S. R., Vilnis, L., Vinyals, O., Dai, A. M., Jozefowicz, R., and Bengio, S., (2015). Generating sentences from a continuous space. *arXiv preprint arXiv:1511.06349.*
